# Reduced and stable feature sets selection with random forest for neurons segmentation in histological images of macaque brain

**DOI:** 10.1038/s41598-021-02344-6

**Published:** 2021-11-26

**Authors:** C. Bouvier, N. Souedet, J. Levy, C. Jan, Z. You, A.-S. Herard, G. Mergoil, B. H. Rodriguez, C. Clouchoux, T. Delzescaux

**Affiliations:** 1 CEA, CNRS, MIRCen, Laboratoire Des Maladies Neurodégénératives, Université Paris-Saclay, Fontenay-aux-Roses, France; 2 Witsee, Paris, France; 3 Service de Médecine Physique Et de Réadaptation – APHP Hôpital Raymond Poincaré, Garches, France; 4 UMR 1179, Handicap Neuromusculaire – INSERM-UVSQ, Montigny le Bretonneux, France; 5 Shaanxi Key Laboratory for Network Computing and Security Technology, School of Computer Science and Engineering, Xi’an University of Technology, Xi’an, China

**Keywords:** Computer science, Machine learning, Image processing

## Abstract

In preclinical research, histology images are produced using powerful optical microscopes to digitize entire sections at cell scale. Quantification of stained tissue relies on machine learning driven segmentation. However, such methods require multiple additional information, or features, which are increasing the quantity of data to process. As a result, the quantity of features to deal with represents a drawback to process large series or massive histological images rapidly in a robust manner. Existing feature selection methods can reduce the amount of required information but the selected subsets lack reproducibility. We propose a novel methodology operating on high performance computing (HPC) infrastructures and aiming at finding small and stable sets of features for fast and robust segmentation of high-resolution histological images. This selection has two steps: (1) selection at features families scale (an intermediate pool of features, between spaces and individual features) and (2) feature selection performed on pre-selected features families. We show that the selected sets of features are stables for two different neuron staining. In order to test different configurations, one of these dataset is a mono-subject dataset and the other is a multi-subjects dataset to test different configurations. Furthermore, the feature selection results in a significant reduction of computation time and memory cost. This methodology will allow exhaustive histological studies at a high-resolution scale on HPC infrastructures for both preclinical and clinical research.

## Introduction

Preclinical studies are crucial to develop and validate novel therapeutic strategies in translational research^1^. Techniques assessing the relevance of a new therapeutic drug range from behavioral studies to tissue analysis which can be qualitative (visual analysis) or quantitative through measurements. Histology is the science studying tissues of animals using microscopy. In the context of brain development, aging and neurodegenerative diseases, histology enables a better understanding of the mechanisms involved using specific biomarkers^2,3^. To decipher these mechanisms, biologists usually perform analysis mostly based on manual quantification of stained tissues such as stereology^4^ or manual segmentation. For whole organ analysis, exhaustive quantification is at least challenging, at most impossible^5^, as these techniques are limited to a specific region or subregion. Furthermore, modern optical microscopes have increased the difficulty of exhaustive analysis by allowing the digitization of histological whole slide images—WSI—at a sub-micrometric in-plane resolution. Resulting images reveal small biological objects—such as individual cells—but consequently their sizes have dramatically increased from megapixels (Mp) to terapixels (Tp).

In this context, the automated quantification of histological images is a prerequisite to speed up data processing and to reduce human workload. Such automation increasingly relies on supervised machine learning (SML) algorithms^6–11^. SML methods are generally split in three main steps: (1) learning, to fit a model with a learning dataset, (2) validation, to evaluate and validate the classification quality of the entire test dataset with the training model and (3) generalization, to use the validated model to analyze an extended set of data. In the past few years, Deep Learning (DL) has allowed significant progress in image segmentation quality^11–13^. However DL are often described as “black boxes” which is a drawback for legally constrained settings such as preclinical and clinical ones^13^. Moreover these methods imply a high level of complexity that prevents end-users (biologists and physicians) from adopting them on the one hand because of the complexity of their implementation (mathematical and computer science skills) and on the other hand because of the lack of intelligibility of the models produced (large neural networks constituted of millions of parameters). Furthermore, DL requires large training databases to reach high segmentation quality compared to SML methods and such databases are difficult, tedious and time-consuming to produce^13^. Moreover a slight corruption of the learning dataset can lead to a high misclassification rate^14^.

SML methods rely on handcrafted information—the features—priorly extracted from raw images. Different kinds of features have been proposed to characterize objects from pixels to whole images such as colorimetric^15^, textural^16^, morphological^17^ and architectural features^18,19^. For this reason the number of features ranges from hundred to several thousands^16,18,20^, dramatically increasing the amount of data to be processed and handled. In the context of virtual microscopy, traditional SML methods are not suited to process petapixels (Pp) images in a short period of time, even using an up-to-date individual workstation or High Performance Computing (HPC) resources^21^.

To overcome this issue, feature selection algorithms (FSA) can be used to reduce the number of features to a small and informative subset of features^22,23^. The main drawback of FSA is the lack of selection stability. These methods rely on the consistency of the results obtained through several executions of the same feature selection algorithm using different data. FSA stability is closely tied with the peaking phenomenon^24–26^. Efficient feature decimation could reduce computational burden by decreasing the quality of segmentation. However, it also increases the possibility of convergence to a learning-based-specific feature subset. To overcome this overfitting issue, an increasing number of methods have been proposed to provide stability measurement criteria, validation methodology and bias correction^24,27–29^. However, none of these methods provide a gold standard reference or a generic framework to compare the different feature selection algorithms to each other.

In this paper, we propose an original framework to quantitatively select and evaluate optimal vectors of the most common handcrafted features to segment histological images, based on the quality and robustness of classification results. We chose to carry out this study using Weighted Random Forests method (WRF) which is a robust and widely used method in this field. An adapted brute-force strategy is proposed to evaluate results of thousands of feature combinations through a two-steps procedure. First, feature families—an original intermediate feature pool between spaces and individual features we introduced—are compared and ranked according to their performances to operate a first segmentation. In this context, we propose an original metric to quantify stability of features, families and spaces to perform an optimal segmentation: the feature Median Position Value (fMPV). Then the individual features are selected from pre-selected feature families following an aggregation-based algorithm to derive an optimal vector of features limited in size with a high segmentation quality. In two preclinical neuroscience studies, we highlight that those stable selected subsets of features associate complementary properties such as combination between edge and blob detectors. Furthermore, we investigate these properties of colorimetric and textural feature space, and compare them with the described properties in the literature. The proposed methodology is validated with two stained macaque central nervous system anatomical regions: the brain—a group study—and the brainstem – one subject -. We also compared the resulting segmentation quality with U-Net^11^ that constitutes an acknowledged reference in the field of Deep Learning segmentation. To assess scalability and performance of the proposed methodology, it is tested on various computer resources, ranging from individual workstation to HPC cluster.

## Part I: material and methods

### Histological datasets

#### Ethical statement

All experimental protocols were approved by CETEA (Comité d' éthique en expérimentation animale) n°44 and the Ministry of higher education, research and innovation (MESRI). The datasets used were histological images of macaque central nervous system sections. Four animals were euthanized by injection of a lethal dose of pentobarbital (Dolethal, Vetoquinol, France). Their brains (*n* = 3) and brainstem (*n* = 1) were extracted according to European ethics rules. All animal studies were conducted according to French regulations (EU Directive 2010/63—French Act Rural Code R 214-87 to 131). The animal facility is authorized by veterinarian inspectors (authorization n° B 92-032-02) and complies with Standards for Humane Care and Use of Laboratory Animals of the Office of Laboratory Animal Welfare (OLAW—n°#A5826-01). The study is reported in accordance with ARRIVE guidelines.

#### Dataset description

**Figure 1:**
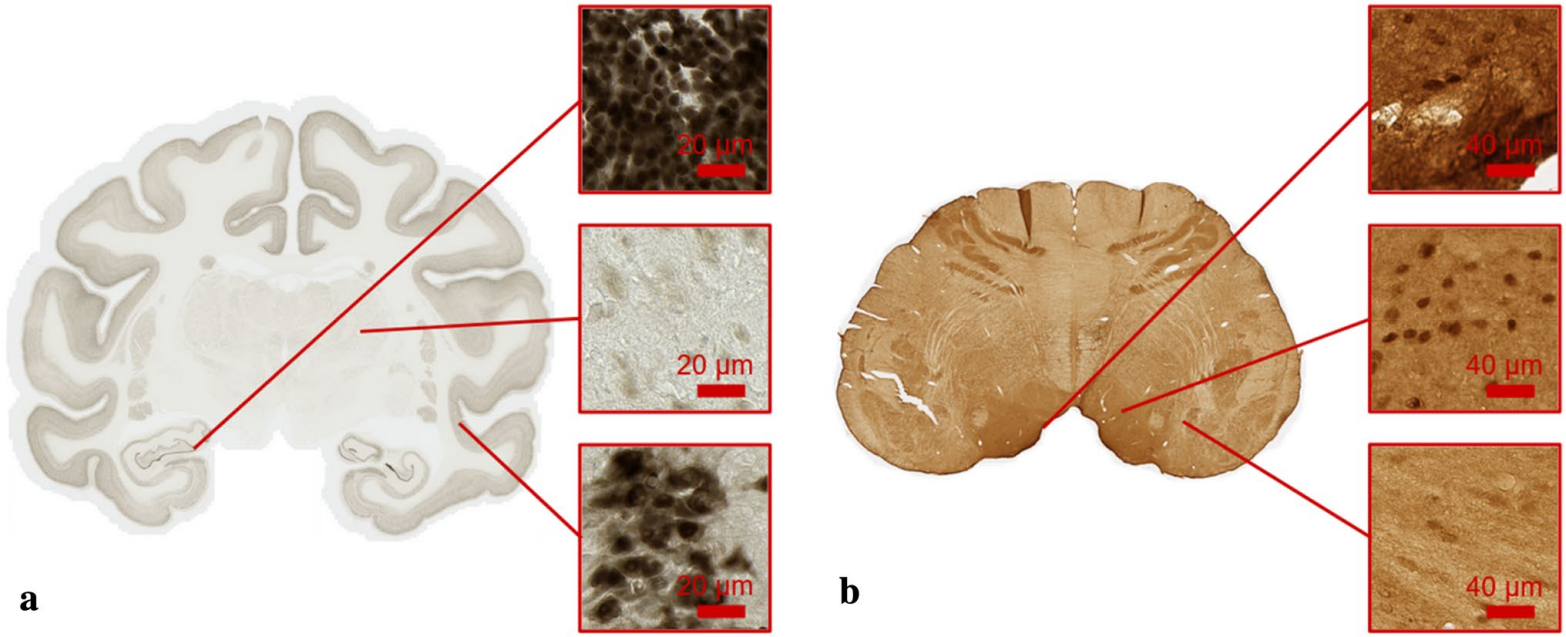
Examples of histological images processed. (**a**) Whole coronal brain section from NeuN_1 stained with DAB-Ni NeuN with three images of 512 × 512 pixels representing the diversity of neuron density and intensity. (**b**) Whole transversal brainstem section stained with DAB anti-Phox2B illustrating the complexity of the non-specific staining to be studied.

Three brains of 9, 6.5 and 5 years old healthy male macaques were cut into 8 series of 40-µm-thick coronal Sects. ^30,31^. For the first brain, one series of 133 sections was produced and stained with DAB-Ni Neuronal Nuclei (NeuN) using a standardized protocol ensuring reproducible staining among sections. Only one section was produced and stained for the two other macaque brains. All the sections were digitized using a Whole Slide Imaging (WSI) bright field virtual scanner (Axio Scan.Z1, Zeiss), with a × 20 magnification factor (in-plane image resolution of 0.22 × 0.22 µm). Each digitized slice weighed approximately 40 Gigapixels (Gp) and exhibited various levels of neuronal density (Fig. 1a).

Three ground-truth datasets were created by extracting a hundred sample images (512 × 512 pixels) in a predetermined number of anatomical regions of the brain spread through the middle sections for each macaque. Based on biologist expertise, the selected sections are representative of density and intensity variability of DAB-Ni NeuN staining. This ground-truth dataset was manually segmented by an image processing expert into four classes: stained tissue (neurons), unstained tissue, background (no tissue) and artifacts (non-specific staining concentration). Learning and test datasets were defined from the dataset according to three rules: (1) each dataset was extracted from middle sections in 3 subjects, (2) each dataset was extracted from the same anatomical regions and (3) each dataset was balanced through their number of images. Following these rules, balanced learning and test datasets were produced (512 × 512 pixels images). For the 9 years old macaque (NeuN_1), 54 and 46 sample images were respectively extracted for the learning and the test datasets (the entire datasets are represented in Fig. 5S). For the last macaques (NeuN_2 and NeuN_3), 50 and 50 sample images were respectively extracted in two balanced datasets (a learning and a test datasets). The pooling of these three datasets is called NeuN_pool and is composed of 300 sample images.

The brainstem of a 5 years old healthy male macaque was cut into 8 series of 40-µm-thick transversal Sections^32^. One series of 60 sections was used for DAB anti-Phox2B labelling. This nuclear marker identifies neurons involved in vegetative functions and automatic breathing control^33,34^. All the sections were digitized using a WSI bright field virtual scanner (Scanner Aperio AT2, Leica biosystems), with a × 20 magnification factor (in-plane resolution of 0.5 × 0.5 µm). Each digitized slice weighed approximately 2 Gp (Fig. 1b).

Contrary to DAB-Ni NeuN staining, the DAB anti-Phox2B was nonspecific and neurons of interest were uncommon. Manual segmentation was therefore difficult to perform as there were no existing atlas to guide neurons of interest localization and distribution^32^. A ground-truth dataset was constructed by extracting 212 sample images (256 × 256 pixels) randomly selected in different regions of the brainstem. Several images were randomly picked on specific areas selected by a pathologist. Then a manual selection was performed on these images to keep the similar proportion of four classes between learning and test datasets. Sample images were manually segmented by a pathologist in four classes: neuron (specific anti-Phox2B staining), tissue (unstained tissue and non-specific staining), background (no tissue) and artifacts (non-specific staining concentration). The entire datasets are represented in Fig. 6S.

### Design of initial feature vectors

SML methods need a large quantity of raw data and features to ensure high precision and specificity of stained tissue segmentation^10,21^. A number of features are available to quantify different properties of images, such as color or textural aspect. To extract the initial features at the pixel scale, four different colorimetric spaces, the local mean, the local variance and three different textural spaces were considered. These features were selected in the most common feature spaces used in biomedical image analysis literature^15,16,18^.

#### Colorimetric feature spaces

A color space is a vectorial modeling of color, and is generally characterized by two properties: linearity and similarity with human perception of color^15^. Color spaces generally have three components expressing different color properties, such as luminance and hues. RGB (Red, Green and Blue hue intensities, respectively) is the most common color space. It is linear and easy to acquire with simple optical color filters. CIE XYZ is a linear transformation of RGB color space, and corresponds to human color perception. Y stands for the luminance, Z the human blue hue perception and X a linear combination of green and red hues. HSV and CIE L*a*b* are non-linear transformations of RGB and CIE XYZ, respectively. H is the hue, S the saturation (color strength) and V the value (color darkness). L* is the luminance, a* red to green hues and b* blue to yellow hues. Due to their non-linear transformation, HSV and CIE L*a*b* are unstable at low saturation levels. A slight change in saturation can result in a significant alteration in the transformed value^14^. However, these spaces better describe hue changes compared to RGB and CIE XYZ in histological images and color images in general^15^.

#### Textural feature spaces

Haralick and colleagues proposed for the first time a descriptor to characterize and extract textural information^35,36^. The goal is to integrate statistical organization of gray intensity values in a limited square window of the processed image. Four Gray Level intensity Co-occurrence Matrices (GLCM) are computed to describe the organization of gray level intensities at 4 different angles in a given neighborhood. The 4 angles of the 8-connectivity are generally used. For each matrix, 16 features are computed. Recent work has demonstrated that only 4 of the 16 features are valuable to describe Haralick textural information: the angular second moment, the correlation, the contrast and the variance^16,37^. Haralick features have several drawbacks limiting their use. First, the algorithm complexity makes Haralick computation-expensive in time and memory. Second, the number of components to store is 16 at a given scale, making it difficult to use in the context of massive histological images^35,36^^.^

Gabor filters are linear filters used to describe textural information of an image^38,39^. Gabor filters impulsional response is the multiplication of a sinusoidal wave with a gaussian function. A frequency in a specific direction is convolved with a local region in the image. The convolution is the Gabor filter response. A Gabor filter has 5 hyper-parameters, resulting in a number of different responses corresponding to the multiplication of the cardinality of the different hyper-parameters. The multiplicity of responses provides a detailed description of the textural information and is used in a wide range of applications, from text analysis^39^ to tumor detection^16^. However, the number of responses (from dozens to hundreds) is an issue for massive histological images multiplying by the same number of data to handle^16,18,40^.

Local Binary Pattern (LBP) is one of the most concise textural features available^41^. Ojala proposed a unique value to describe gray level patterns around a pixel at a specific scale. All gray level intensities of the pixels lying in a circle around a given pixel are subtracted to the central gray value intensity. The values are then vectorized and thresholded. If the value is positive it becomes 1 else 0. The binary vector is then converted into a natural integer. Several variants of LBP exist but one of the most popular versions is Rotation Invariant Uniform Local Binary Pattern^42^. The binary vector is minimized to make LBP Rotation Invariant. Then uniform patterns can be detected. An uniform pattern is defined as a pattern containing only two changes in zeros and ones. Uniform patterns describe the majority of fundamental patterns in an image, including, but not limited to, edges, lines and corners^42^. LBP has low computational complexity and low memory consumption. It is particularly adapted to textural information computation in big data^41^^.^

#### Initial feature vector

The initial feature set was designed with the different spaces detailed previously. The initial feature vector was composed of 114 elements including: 4 color spaces (RGB, HSV, CIE XYZ, CIE L*a*b*), local mean intensity, local variance intensity and 3 textural spaces (Gabor, Haralick, LBP) at 4 different radiuses (Table 1). For NeuN images, the estimated diameter of neurons ranges from 2.5 to 15 µm^43^. To avoid loss of neuronal information, the radius of the structuring element for mean and variance computation was set to 2.2 µm. Likewise, the first three structuring element radiuses for textural spaces were chosen to match the neurons size range (2.42, 8.8 and 14.96 µm). The last structuring element radius was set to twice the maximum radius of neuron (30 µm) to determine maximal radius to investigate. The different gaussian parameters of Gabor filters were set to have an ellipsoid kernel with a size smaller than the minimum neuron size (aspect ratio equal to 0.38 µm and gaussian standard deviation equal to 0.5 µm). The variable parameters were the wavelength and the orientation of the Gabor filter. The four main directions of 8-connexity were chosen (0°, 45°, 90° and 135°). Wavelengths were chosen to sample texture between 2 and 20 pixels in order to not exceed the gaussian ellipsoid support (0.55, 1.1, 2.2, 4.4 µm). Both real and imaginary parts were computed.

**Table 1:** Sum up of initial feature vector parameters for NeuN and anti-Phox2B staining.

Feature space	Parameters for NeuN (in pixels/degrees)	Parameters for anti-Phox2B (in pixels/degrees)
Mean and Variance images	10	5
LBP	11 ; 40 ; 68 ; 134	5 ; 10 ; 20 ; 40
Haralick (GLCM)	11 ; 40 ; 68 ; 134	5 ; 10 ; 20 ; 40
Gabor filter	Aspect ratio: 1.5 Standard deviation: 2 Phase: 0° Orientations: 0° ; 45° ; 90° ; 135° Wavelengths: 2.5 ; 5 ; 10 ; 20	Aspect ratio: 1.5 Standard deviation: 2 Phase: 0° Orientations: 0° ; 45° ; 90° ; 135° Wavelengths: 2.5 ; 5 ; 10 ; 20

The initial feature set for anti-Phox2B staining was designed to be similar to the initial feature set of NeuN staining. The pixel-size of the neurons detected by anti-Phox2B were equivalent to NeuN. Because of the lower in-plane resolution, the different radius in pixels were increased accordingly. With the same rationale as NeuN staining, the four structuring element radiuses were then chosen at 2.5, 5, 10 and 20 µm. Gabor parameters were set to the same as NeuN staining.

### Segmentation using weighted random forest

Random Forest (RF) is a method allowing efficient segmentation at a low computational cost on gigapixels images^8^. Furthermore it is one of the most used algorithms in the context of virtual microscopy^8,44–46^. RF is composed of a set of fully grown decision trees which are trained based on a bootstrap of the training database and with a randomized vector of features. Each tree provides a classification decision and the majority of the decisions prevails. One of the main properties of RF algorithms is nonlinear boundary fitting, making it particularly relevant to detect specific against nonspecific staining. Weighted Random Forest (WRF) algorithm^47^ uses weights to strengthen minority classes of interest in a strongly unbalanced dataset. The weights are adjusted independently from the training process. In the case of a minority class of interest, WRF offers a valuable alternative to RF. In the proposed study, WRF was implemented using Scikit-learn^48^. The three main parameters to adjust are the number of trees, the maximal depth of the trees and the vector of weights for the classes considered. Each parameter conditions an aspect of WRF performance: the first one the stability of the final decision and noise reduction and the second and the third the overall accuracy but also the overfitting risk.

In our context, WRF was used to segment stained tissue. For each study and using the same initial feature vector, WRF was optimized and then evaluated using F-Score criterion to compare automatic and manual segmentations^49^. The number of trees was set to 100. The optimization of the decision tree depth was performed first and subsequently the weight of the class of interest. Since F-Scores quantify segmentation quality, we considered that a F-Score superior to 0.8 represented a good segmentation. WRFs were then validated using two-fold-cross-validation by swapping learning and test datasets. The results of both evaluation and validation were used to guide the proposed feature selection methodology.

### Brute-force selection

#### General description of the methodology

The proposed methodology of selection aimed at finding a reduced, stable and relevant feature subset from an exhaustive initial feature vector. A Brute-force searching and an original criterion, the feature Median Position Value (fMPV) were proposed to fulfil these requirements. The main benefit of a Brute-force searching is the exhaustive representation of possible solutions^50^ and the certainty to identify the optimal combination of features. However, the generation and test of all possible solutions is impossible to compute due to the tremendous number of combinations to consider.

**Figure 2:**
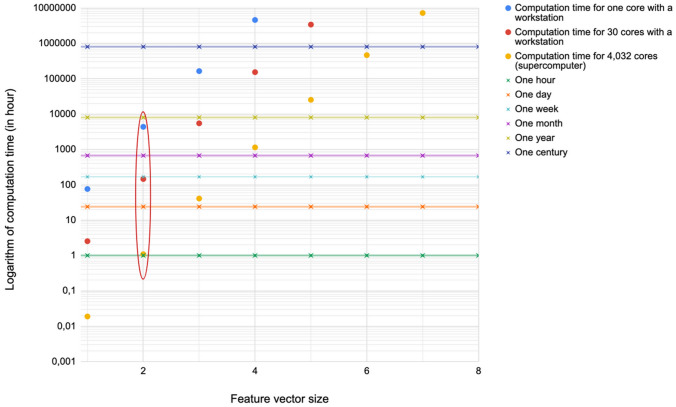
Computation time (logarithmic scale) for 114 features, different computational configurations and according to vector size (n = 2). The horizontal lines represent different time scales (from an hour to a century) and an averaged computation time for one combination was estimated to be equal to 40 min. The circled points represented acceptable processing times for the three different computation configurations and lead to a maximal vector size of 2.

With the initial feature vector (114 features), the total number of explored subsets would reach $2.1{0}^{34}$. Given that a subset feature requires about 40 min to be processed with WRF, exploring the whole subsets was impossible, even with a supercomputer. Consequently, we proposed to limit the explored combinations in size. Figure 2 presents the processing times estimated according to different numbers of computational cores and sizes of feature vectors ranging from 1 to 8 features. In HPC context, the use of 4,032 cores of a supercomputer was tested as well as 30 cores for a workstation. Processing times superior to a week for a workstation or several days for a supercomputer were considered as too prohibitive. The main reason for these choices was the time of utilization of supercomputers thought to be reasonable to produce and confirm the results of the methodology. Based on these considerations, the maximal vector size chosen was 2 as highlighted in Fig. 2.

**Figure 3:**
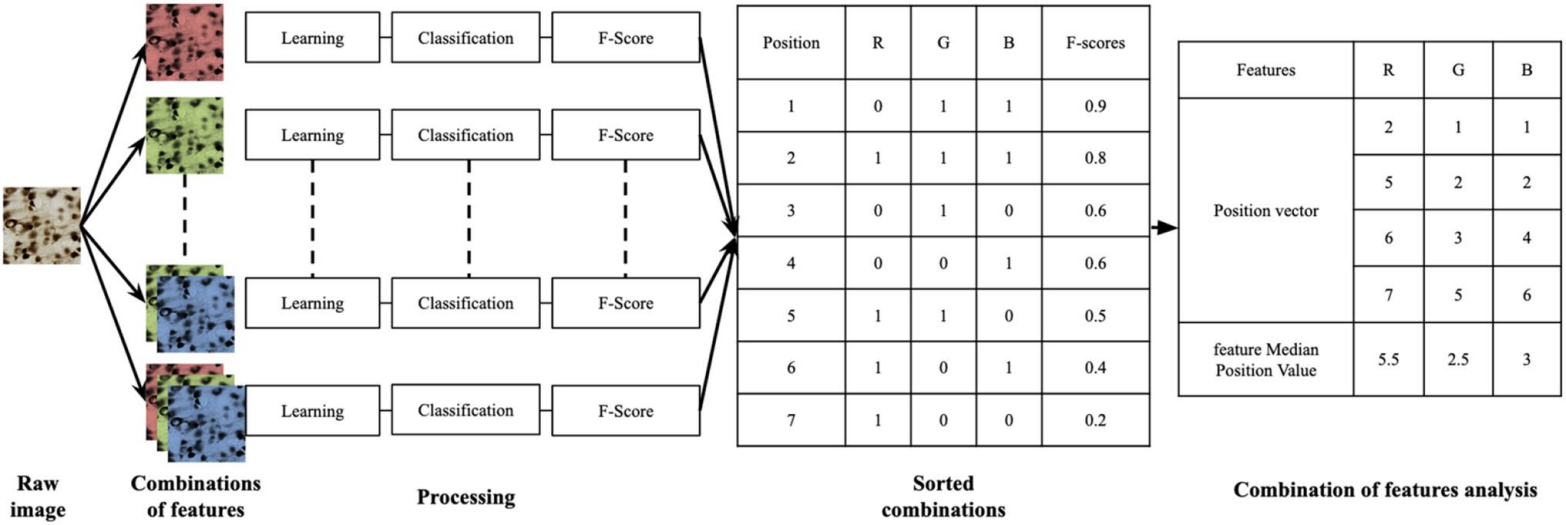
Example of Brute-force searching using three features R (Red), G (Green) and B (Blue). ‘0’ represented the absence of the feature and ‘1’ its presence in the sorted combinations table. Each feature had its own position vector and was scored with fMPV.

The F-Score for the class of interest was computed for the WRF method with each generated combination. The F-Scores and the corresponding feature combinations were then concatenated into a single table and sorted in decreasing F-scores order. For each individual feature, a vector of the different positions in which it appears in the sorted list was extracted. The position variable can be considered as a penalty (low value for the best segmentations and vice versa) and its frequency of appearance in the sorted list is a reliable index of its relevance to produce a good segmentation. Compared to approaches that limit themselves to finding one optimal combination, our approach allows us to estimate in a more general way the dispersion of the positions of each feature in all the combinations where it is present, but also to compare the individual features with each other. In order to synthesize the distribution of a given feature in the form of an index, we have chosen to calculate the median position value for each feature (fMPV) (Fig. 3). This approach makes it possible to generate a secondary ranking of the features according to their ability to produce a good segmentation. Moreover, it is possible to use this criterion at different scales (spaces, features families and individual features). To our knowledge, the use of the position variable of the features combined with the calculation of the median value to characterize their distribution in a large set of combinations is original.

Due to large processing times, it was not possible to explore entirely the space of feature combinations. Thus a limitation in the size of the explored vector was proposed. To enhance furthermore our capacity to analyze large amounts of combinations, we introduced an original way of pooling features together: the feature families. The first selection step concerned the feature families and allowed a significant reduction in the number of processed features to investigate. This reduction allowed an increase in the limit of the feature vector size for a second selection step.

##### *First step: family feature definition and selection*

Textural families were defined from textural spaces as non-oriented groups of features sharing the same structuring element radius (i.e. spatial scale of computation) and the same descriptive properties. Thus, a specific Gabor family included the four Gabor filters with only orientation of the sinusoidal wave hyper-parameter changing. A specific Haralick family was represented by the collection of statistical components in the four directions corresponding to 8-connexity. LBP, Mean and Variance families were their single component. The 114 features were pooled in 4 colorimetric families—which is equivalent to spaces—and 30 textural families (Mean, Variance, 4 LBP families, 8 Gabor families and 16 Haralick families) (Table 2). The cardinality of the textural families defined was similar to colorimetric spaces cardinality.

**Table 2:** Sum up of textural feature families cardinality for different feature spaces.

Textural space	Feature family definition	Cardinality of the families
Haralick feature space	For each GLCM: Angular second moment Correlation Contrast Variance	4 4 4 4
Gabor feature space	For each orientation: Real parts Imaginary parts	4 4
LBP	No subdivision	1

The nomenclature proposed to name features families was fixed as follows: a Gabor family was named by the letter G followed by its wavelength and the letter R for “Real part” or I for “Imaginary part”. A component of a Gabor family was designated by linking the angle to the name of the Gabor family. A Haralick component family was designated by the letter H followed by A for “Angular second moment”, Corr for “Correlation”, Con for “Contrast” or V for “Variance” and a number for the radius of the structuring element in pixels. For example, G_20_R would be the real part of the Gabor filters with a wavelength of 20 pixels.

The relevance of a feature family was defined based on two properties: (1) the fMPV of all constituting features of a family must be inferior to the mean fMPV of all individual features and (2) the fMPV of its corresponding space must be inferior to the mean fMPV of all spaces. Both properties led to less than a quarter of the 114 features kept. Since this step was performed in direct and cross-validation, two sets of relevant families have been defined. The selected families were the common families of both sets. The constitutive features of these families formed a new initial feature vector for the second selection step.

##### *Second step: feature selection*

**Figure 4:**
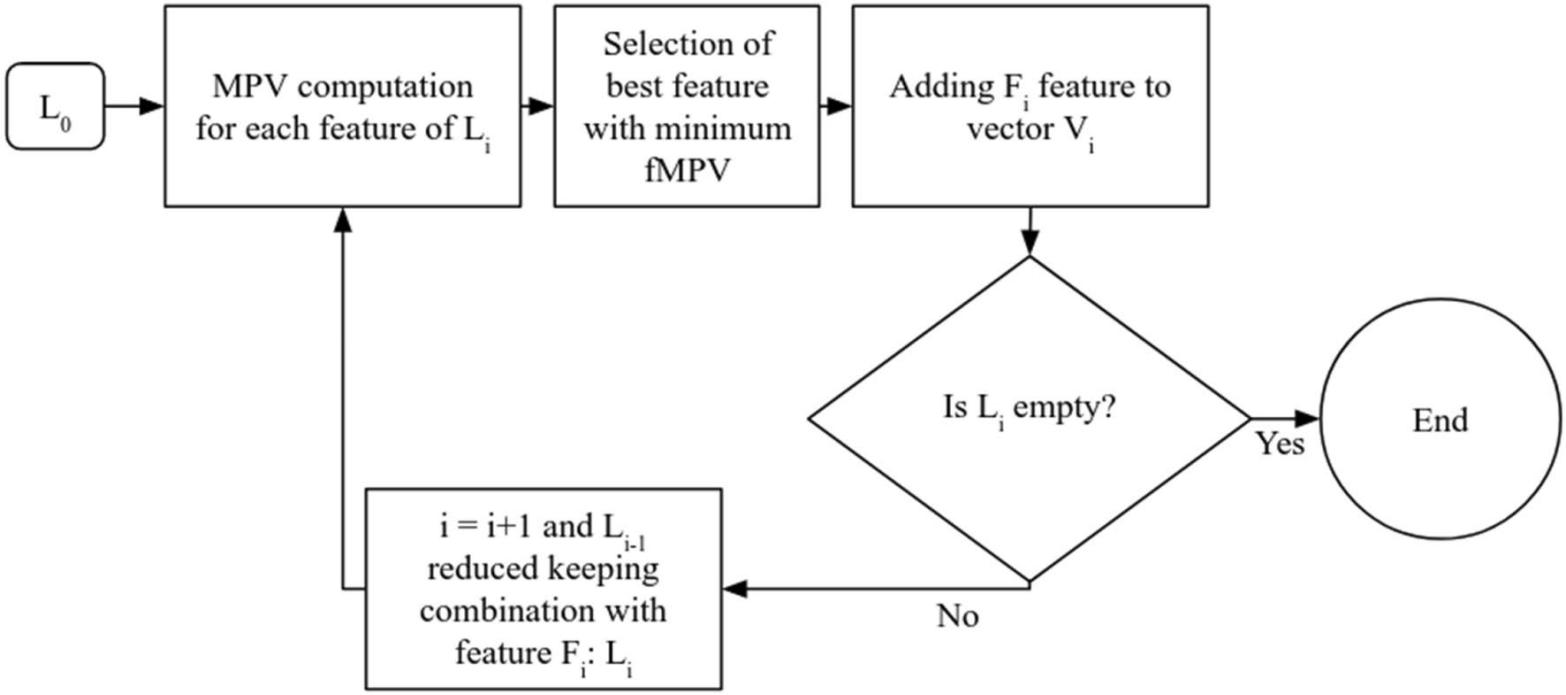
Flowchart of the proposed feature aggregation scheme (second selection step). *L*_0_ was the list of all the combinations tested during brute-force searching based on feature families selected. *L*_*i*_ represented the list of remaining combinations at iteration *i*. *F*_*i*_ represented the feature with the lowest fMPV at iteration *i*. *V*_*i*_ represented the feature vector at iteration *i* (for *i* = 0, *V*_*i*_ is empty)^51^.

The optimal feature vector was determined iteratively by selecting at each round the optimal feature minimizing the fMPV^51^, and by considering in the following iteration only the combinations including the previous selected features (Fig. 4). This strategy rapidly led to a decrease in the number of combinations to be investigated until no features remained. The selected vector was the feature vector with the best F-Score among all iterations and which minimized the vector size. The selection was performed on a limited vector size as the first step (*n* > 2). This limit was determined after the first selection depending on the number of features previously selected. The selection was also performed in two-fold-cross-validation to verify the independence to the learning and validation datasets of the selected vector^25^. If both vectors were unsimilar, the vector with the highest F-Score was therefore selected.

##### *Generalization step*

Using the proposed strategy, a reduced and stable feature vector was selected for each stain. Subsequently, a whole high-resolution digitized organ (Part I) from each staining was segmented using both optimized subsets and WRF within a distributed CPU computation environment. For the macaque brains, only the first one was processed (NeuN_1). Since all feature values were coded in float32 bits, a reduction of the feature vector led directly to a proportional reduction of the memory used. Combined with parallelization, the segmentation processing time was reduced and measured according to the number of cores and the size of the features set determined. The different results are presented in the next part. 

### Comparison with U-Net

U-Net is a DL method aiming at segmenting images at a pixel-scale. The architecture is based on auto-encoder layers coupled with convolutional neural networks^11^. Convolutional layers can be divided in two types: the encoders extracting convolutional features through multiple resolution levels and the decoders synthesizing the segmentation in all the encoded features map starting at the lower-resolution level. ReLU and pooling layers are included in the architecture between the convolutional layers. Several hyperparameters are tunable: the size of convolutional kernel, the feature root (number of filters in the starting encoded layer), the size of the pooling filters, the number of encoding/decoding layers, the batch size, the dropout rate and the number of learning epochs.

To compare the results of the proposed method, the network tested was composed of 3 encoding/decoding layers, 3 × 3 convolutional filters, 2 × 2 max pooling filters, a feature root of 16, a batch size of 2, 0.1 of dropout and 1,000 epochs. The loss function was the binary cross-entropy^11^. The network has been trained with the same learning and testing datasets used for the WRF. Contrary to Falk et al*.*^11^, no data augmentation was used for this test to allow fair comparison with the same datasets. Direct and cross validation were performed. The results are presented in the next part.

### Computational environment

The WRF and feature selection codes were developed and integrated in the BrainVISA collaborative software platform (http://brainvisa.info)]^52^ Software libraries enabling partial Input/Output access and distributed CPU computation (somaWorkflow^53^) allowed partial reading of large images, fitting the processed data to computational requirements of a HPC environment. Using this framework, each histological section was processed in parallel, reducing the required processing time for pixel-by-pixel feature extraction and segmentation. The implementation of U-Net was realized with Tensorflow and Keras^54^, two Python API for DL algorithms.

Computations of WRF and feature selection were performed on two different Information Technology (IT) infrastructures. A workstation with Ubuntu 14.04 LTS 64-bits on Intel Xeon CPU E5-2630 v3 @ 2.40 GHz × 16 (32 computing cores), 128 GB of Random Access Memory (RAM) and the supercomputer *Irène* of HPC infrastructure Très Grand Centre de Calcul (TGCC) of the french atomic commission CEA (http://www-hpc.cea.fr/en/complexe/tgcc-Irene.htm). *Irène* has 1,656 computing nodes Intel Skylake @ 2.7 GHz (AVX512) with 48 cores and 192 GB of RAM each. The computation of U-Net training and validation were performed on a workstation with Ubuntu 16.04 LTS 64-bits on Intel Core i9-10900X @ 3.7 GHz (32 computing cores), 128 GB of RAM and a NVIDIA Quadro P5000 with 16 GB of V-RAM.

## Part II: Results

### Random forest segmentation

**Figure 5:**
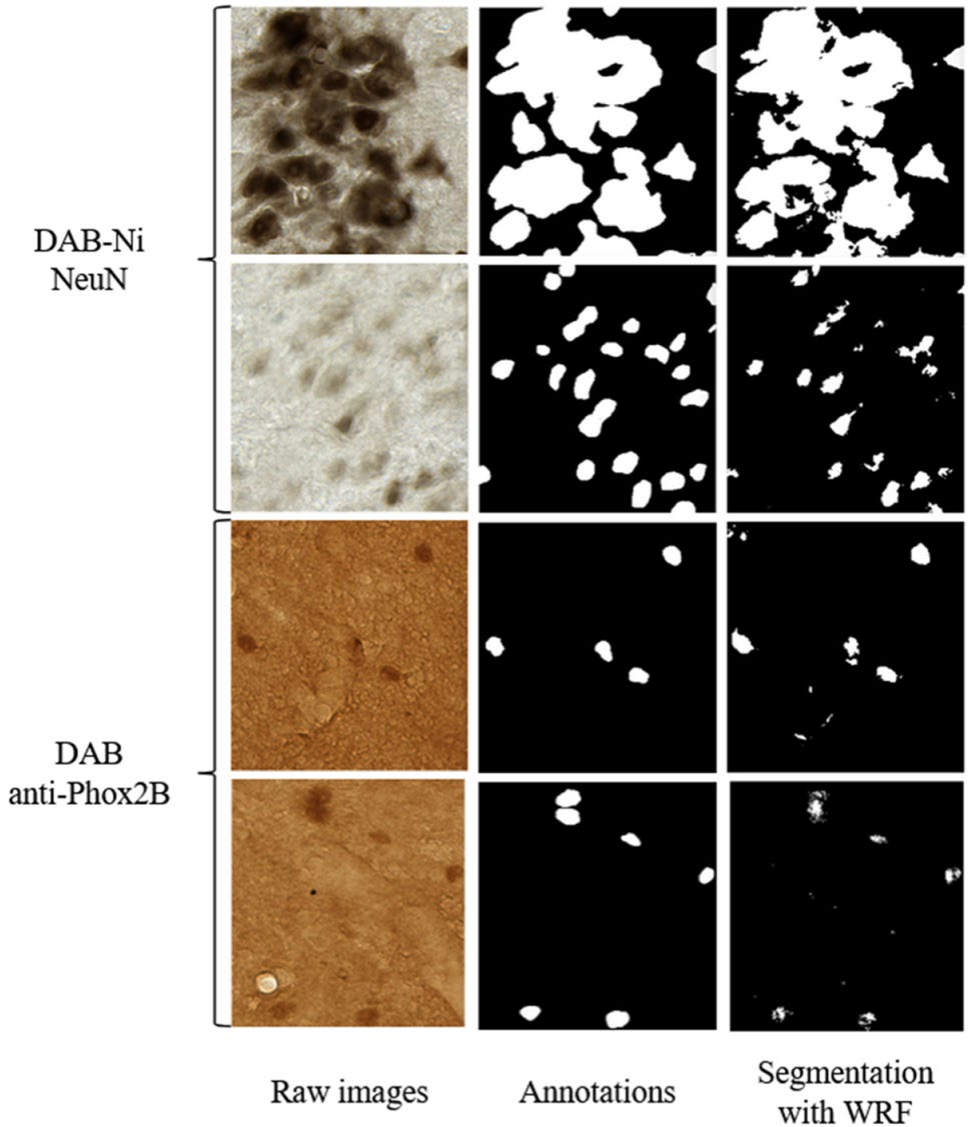
Examples of automatic segmentation obtained with WRF (114 features) for four images from NeuN and DAB anti-Phox2B datasets. Annotations represent corresponding manual segmentations.

**Figure 6:**
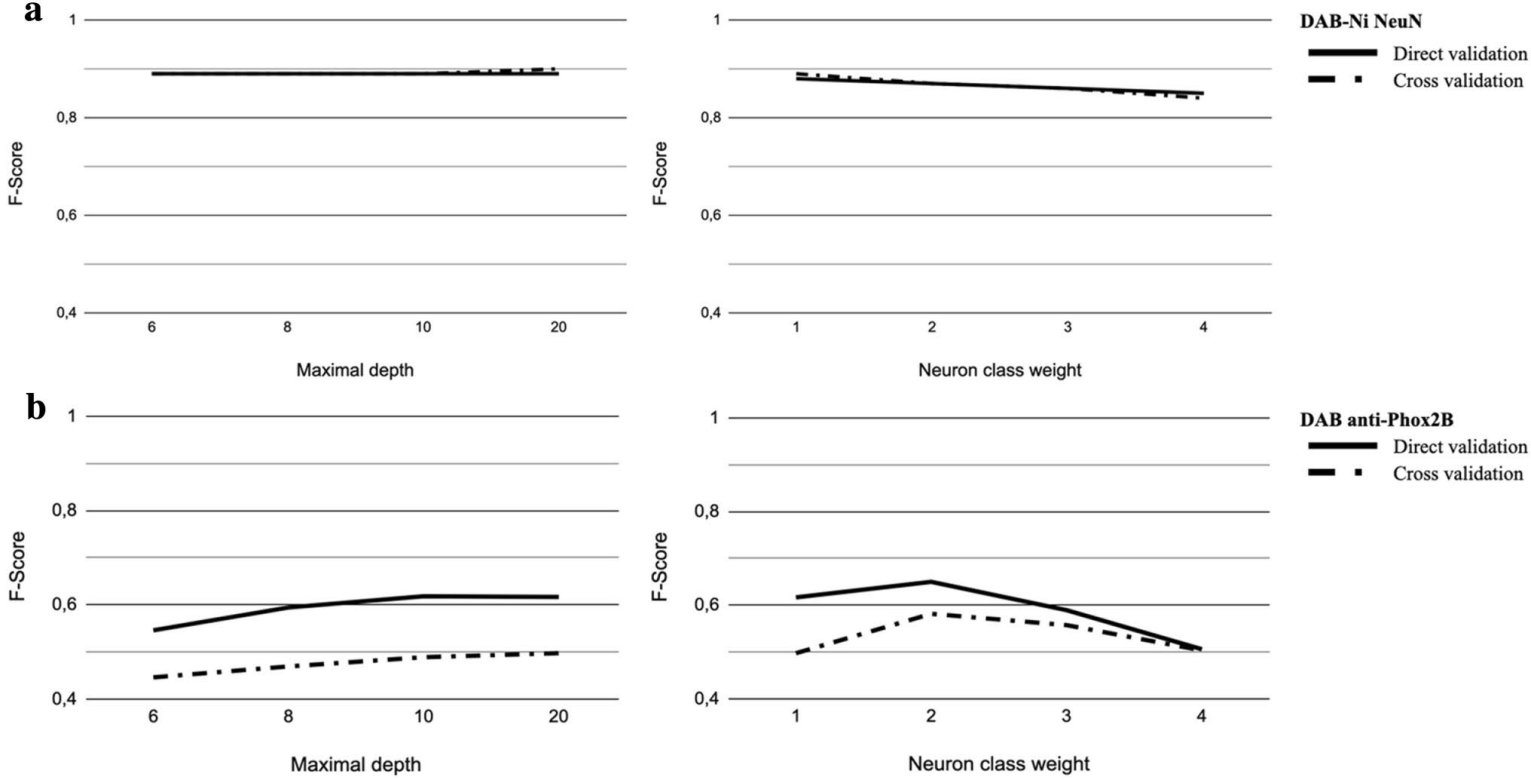
WRF parameters optimization for NeuN (**a**) and DAB anti-Phox2B (**b**). The continuous line represents F-Score in Direct validation. The dotted line represents F-Score in Cross validation. Left: the maximal depth optimization. Right: the neuron class weight optimization.

The NeuN datasets were segmented by WRF with the initial feature vector including 114 features (Fig. 5). F-Score values of 0.88 were obtained in Direct Validation (DV) and 0.89 in Cross Validation (CV) with 100 trees and a maximal depth of four. These scores corresponded to the highest quality of segmentation. Moreover the increase of maximal depth and weight of class “neuron” were tested and did not improve the segmentation quality (Fig. 6a). The DAB anti-Phox2B datasets were first segmented with the same standard WRF settings used for NeuN without optimization. The F-Score values obtained for DAB anti-Phox2B in direct and cross validation were below 0.5 (0.49 in DV and 0.42 in CV) for both, which is insufficient for a correct segmentation.

In direct validation, after optimization, the increase of the maximal depth improved the F-Score to 0.618 at a maximal depth of 10, and to 0.617 at a maximal depth of 20. In cross validation, the F-Scores were 0.489 for a maximal depth of 10, and 0.497 for a maximal depth of 20. From a maximal depth of 10, the WRF became stable as shown in Fig. 6b. The weight of the neuron class was optimized to achieve an optimum F-Score of 0.65 in direct validation and 0.58 in cross validation as shown in Fig. 6b (26% F-Score increase). The resulting weight of the neuron class was twice the weight of the other classes. Therefore, the maximal depth was set to 10, the number of trees to 100 and the weight of the neuron class to 2.0. Examples of automatic segmentation with optimal parameters are shown in Fig. 5.

### Feature family selection (Step 1)

For NeuN staining, selected families were consistent between the two validation conditions (Table 3). Through the 4 datasets studied, 6 out of 10 families were identical between validation conditions and in the same relative order, which represented 21 individual features corresponding to 18.42% of the initial feature set size. The families selected for NeuN_pool dataset were identical to families selected for NeuN_2. Moreover, LBP_11 and Var images were selected for the NeuN_pool dataset and this selection was shared for two thirds of the other datasets. Only LBP_40 and LBP_68 were not selected for the NeuN_pool dataset.

**Table 3:** Summary of the selected families for NeuN datasets.

Selected feature families for NeuN staining	Selected for NeuN_1	Selected for NeuN_2	Selected for NeuN_3	Selected for NeuN_pool
G_20_R	DV / CV	DV / CV	DV / CV	DV / CV
G_10_R	DV / CV	DV / CV	DV / CV	DV / CV
Mean image	DV / CV	DV / CV	DV / CV	DV / CV
CIE XYZ	DV / CV	DV / CV	DV / CV	DV / CV
RGB	DV / CV	DV / CV	DV / CV	DV / CV
HSV	DV / CV	DV / CV	DV / CV	DV / CV
Var image		DV / CV	DV / CV	DV / CV
LBP (11 pixels radius)	DV / CV	DV / CV		DV / CV
LBP (40 pixels radius)	CV			
LBP (68 pixels radius)	CV			

The feature families are sorted according to Fig. 1S, 2S, 9S and 10S. The nomenclature is presented in Material and Methods.

Similar results were found for DAB anti-Phox2B staining (Table 4). Seven families were common between the two validation conditions which represented 24 individual features (21.05% of the initial feature set size). Contrary to NeuN, the relative orders between direct and cross validation were not consistent.

**Table 4:** Summary of the selected families for DAB anti-Phox2B.

Selected feature families for DAB anti-Phox2B staining	Selected for anti-phox2B
LBP (40 pixels radius)	DV / CV
LBP (20 pixels radius)	DV / CV
HSV	DV / CV
LBP (10 pixels radius)	DV / CV
RGB	DV / CV
CIE XYZ	DV / CV
CIE L*a*b*	CV
G_20_R	DV / CV
Mean image	DV
G_10_R	DV

The feature families are ranked through their relevance (more precision in Supplementary Fig. 3 to 4). The nomenclature is presented in Material and Methods.

For both staining, the family selection led to a significant reduction of the number of individual features (approximately one fourth) allowing for a deeper Brute-force searching on the remaining features (increase of the vector size). Figure 11S presents an update of the computation time with the new initial vectors of parameters (resp. 21 and 24 for NeuN_1 and DAB anti-Phox2B). NeuN_2, NeuN_3 and NeuN_pool have respectively 20, 19 and 20 features. Therefore, the estimation presented in Fig. 11S is an upper estimation for these datasets. With the same rationale as previously introduced, the vector size limit was extended to 4 for all datasets. Once feature families were selected for each staining, a second selection step was performed to extend the exploration of possible features combinations.

### Individual feature selection (Step 2)

**Figure 7:**
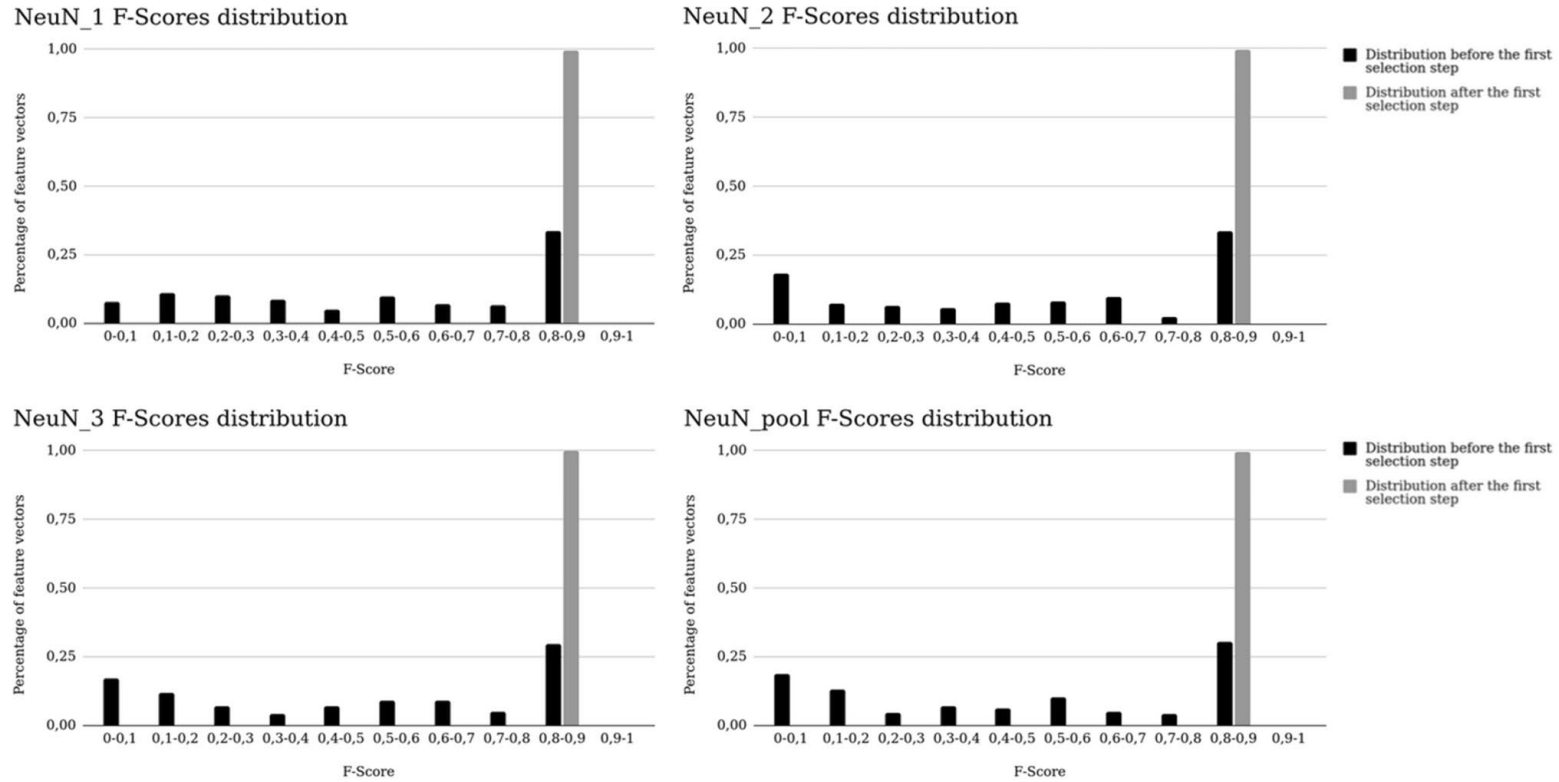
Evolution of the F-Score distribution among all combinations before (in black) and after (in grey) the first selection step for NeuN datasets.

The second Brute-Force search resulted in the evaluation of thousands of combinations (7,546 for NeuN_1, 6,195 for NeuN_2 and NeuN_pool and 5,035 for NeuN_3). After the first selection step, more than 99% of all combinations reached F-Score values higher than 0.8 for all NeuN datasets (Fig. 7). This proportion represented a threefold average increase.

For each dataset, the selected features were similar under DV and CV conditions at each iteration (Table 5). Among selected features, the real part of the Gabor filter with a wavelength of 20 pixels was selected in all selection processes and validation conditions. LBP and Variance images were selected in 3 out of 4 selection processes. For NeuN_1, NeuN_2 and NeuN_3, the selection diverged at the third iteration. The mean final F-Score corresponded to 97% (NeuN_1), 99% (NeuN_2), 98% (NeuN_3) and 98% (NeuN_pool) of the F-Score resulting from the use of the initial 114-sized feature vector. The size of the selected feature vectors were 2 for NeuN_1, NeuN_2, NeuN_3 and 3 for NeuN_pool which is a reduction by a factor of 57 of the initial feature vector.

**Table 5:** Feature selection results for the NeuN datasets.

Iteration number	Validation conditions	Feature vector NeuN_1	F-Score NeuN_1	Feature vector NeuN_2	F-Score NeuN_2	Feature vector NeuN_3	F-Score NeuN_3	Feature vector NeuN_pool	F-Score NeuN_pool
1	DV	LBP_68	0.45	Var	0.62	Mean	0.88	Var	0.55
	CV	LBP_68	0.50	Var	0.63	Mean	0.86	Var	0.55
2	DV	**LBP_68** **G_20_R_135**	**0.86**	**Var** **G_20_R_90**	**0.89**	**Mean** **G_20_R_90**	**0.89**	Var LBP_11	0.58
	CV	**LBP_68** **G_20_R_0**	**0.87**	**Var** **G_20_R_90**	**0.89**	**Mean** **G_20_R_0**	**0.88**	Var LBP_11	0.55
3	DV	LBP_68 G_20_R_135 S	0.85	Var G_20_R_90 Mean	0.89	Mean G_20_R_90 Var	0.89	**Var** **LBP_11** **G_20_R_135**	**0.85**
	CV	LBP_68 G_20_R_0 LBP_40	0.86	Var G_20_R_90 LBP_11	0.89	Mean G_20_R_0 Z	0.88	**Var** **LBP_11** **G_20_R_135**	**0.84**
4	DV	LBP_68 G_20_R_135 S G_10_R_135	0.86	Var G_20_R_90 Mean H	0.89	Mean G_20_R_90 Var S	0.89	Var LBP_11 G_20_R_135 H	0.85
	CV	LBP_68 G_20_R_0 LBP_40 LBP_11	0.87	Var G_20_R_90 LBP_11 S	0.89	Mean G_20_R_0 Z Var	0.88	Var LBP_11 G_20_R_135 H	0.84

Bold vector corresponds to the selected vector. The nomenclature is presented in Material and Methods.

**Figure 8:**
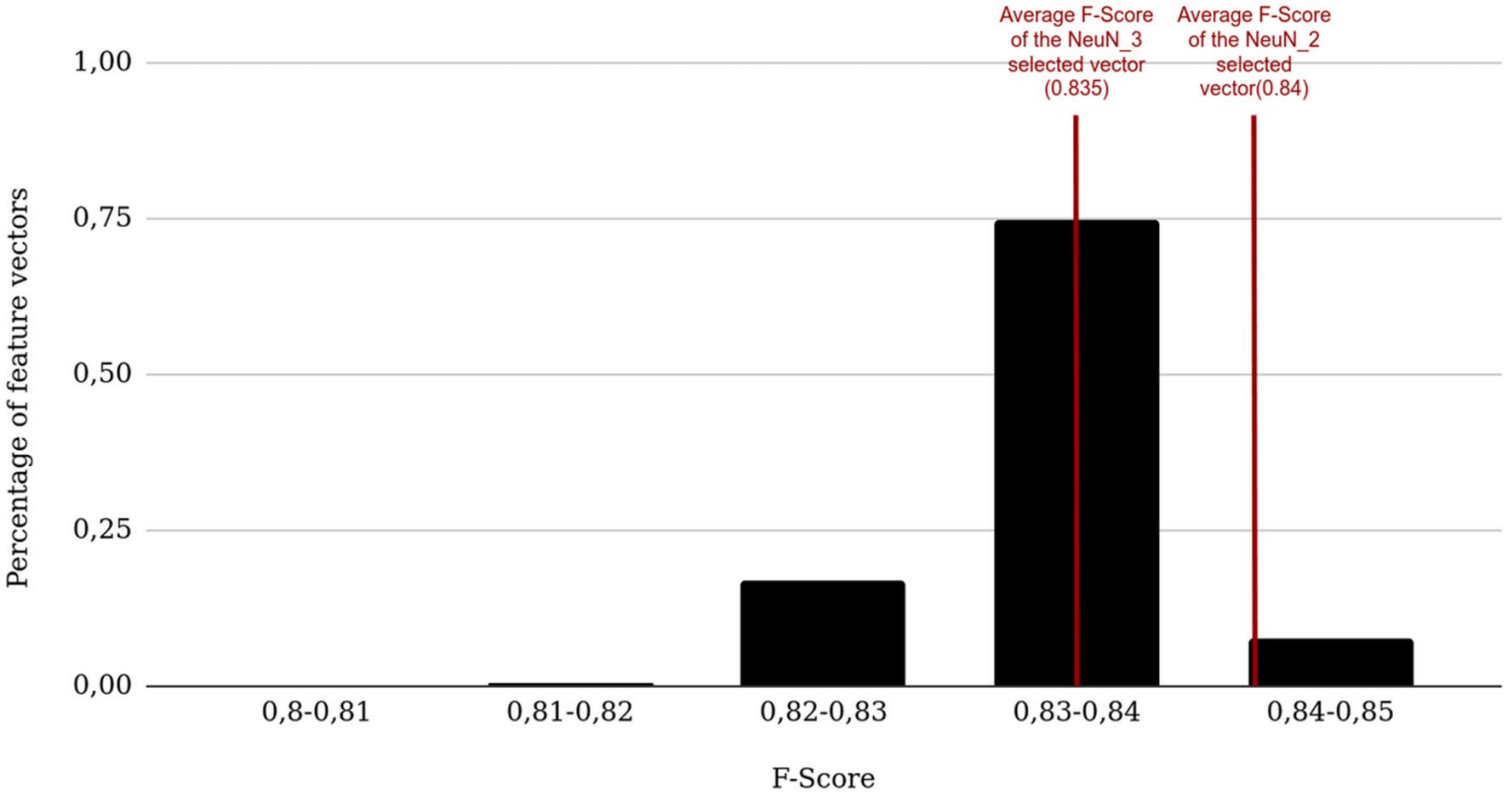
Magnification of Fig. 7 between 0.8 and 0.85 (5 bins for NeuN_pool). The red vertical lines represent the average F-Scores between the two validation conditions (DV/CV) for NeuN_2 and NeuN_3.

LBP_68 was selected only with the NeuN_1 dataset. Therefore, the selected vector of NeuN_1 was not among the evaluated combinations derived from NeuN_pool. The F-Scores of selected vectors of NeuN_2 and NeuN_3 were extracted in the Brute-force searching results of NeuN_pool (Fig. 8). Both extracted F-Scores were very close in the top 50% of NeuN_pool F-Scores.

**Figure 9:**
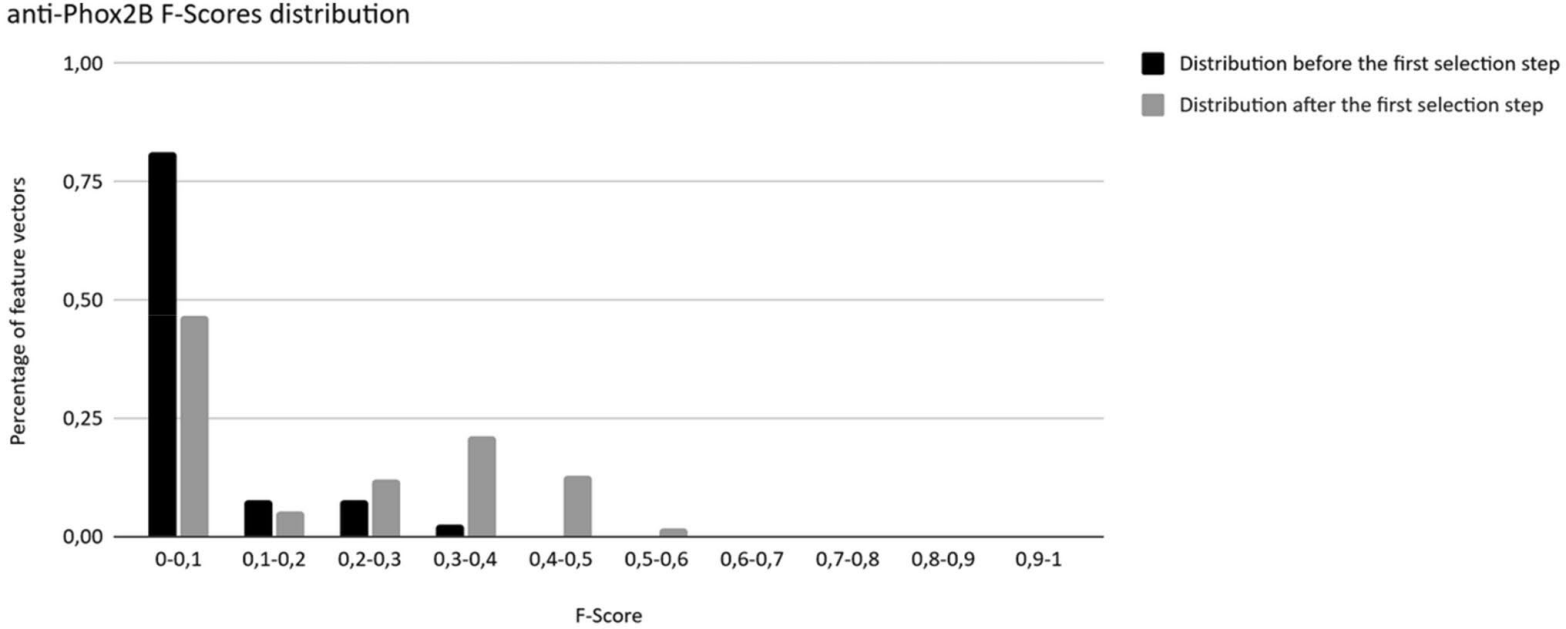
Evolution of the F-Score distribution among all combinations evaluated before and after the first selection step for anti-Phox2B dataset.

A total number of 6,903 combinations were evaluated on the second Brute-force search for the anti-Phox2B dataset. Before the first selection step, 81% of the combinations had a F-Score below 0.1 (Fig. 9). This proportion was significantly reduced to 46% after the first selection step. Only 2.2% of the combinations had an F-Score superior to 0.4 and 36% after the second step. The second selection step improved the F-scores of the selected combinations (shift toward the right part of the distribution). The maximum values of the F-scores were increased from 0.4 to 0.6 between the two steps.

For DAB anti-Phox2B, the first three features selected were consistent between the two validation conditions (Table 6). These features were LBP with a radius of 20 pixels, b* from CIE L*a*b* and a feature included in the real part of the Gabor filter with a wavelength of 20 pixels. Since the last feature was selected with F-Score as criterion (4th iteration corresponding to the maximal size of vector investigated), the first three features were considered as selected with fMPV criterion. For anti-Phox2B staining, the selection algorithm did not reach its main ending condition (F-score still increasing). The selected vector was [LBP_20 ; b* ; G_20_R_90] and its average F-Score between the two validation conditions was 0.5083 which corresponded to 78% of the F-Score resulting in the initial 114-sized feature vector.

**Table 6:** Feature selection results for anti-Phox2B.

Iteration number	Validation conditions	Feature vector	F-score
**1**	DV	LBP_20	0
	CV	LBP_20	0
**2**	DV	LBP_20 ; b*	0.0518
	CV	LBP_20 ; b*	0.0001
**3**	DV	LBP_20 ; b* ; G_20_R_90	0.5083
	CV	LBP_20 ; b* ; G_20_R_0	0.3984
**4**	DV	LBP_20 ; b* ; G_20_R_90 ; LBP_10	0.5277
	CV	LBP_20 ; b* ; G_10_R_0 ; LBP_40	0.4229

Bold vector corresponds to the selected vector. The nomenclature is presented in Material and Methods.

### Generalization with the selected feature vectors

The reduction of the feature vector size coupled with the use of distributed CPU processing brought a significant reduction in physical memory requirement and time computation for both staining. The needed memory was dramatically reduced by a factor of 38 for NeuN_pool and 57 for the other datasets. Using a supercomputer (4,032 cores) with the optimized feature subset, the whole brain (5.3 Tp) was processed 2.5 h hours against 1.5 estimated months without parallelization and feature selection (Table 7). Similarly, the memory needed for the whole brainstem stained anti-Phox2B (120 Gp) was reduced by a factor of 38 and the time consumption was reduced to several minutes against a year without the proposed selection method and parallelization.

**Table 7:** Computation times for feature extraction and classification steps for a whole brain stained with NeuN_1 and a whole brainstem stained with anti-Phox2B.

Staining	NeuN			Anti-Phox2B		
Computer resources	Number of features	Feature extraction processing time	Classification processing time	Number of features	Feature extraction processing time	Classification processing time
1 core	114	~ 6 months	~ 15 years	114	~ 4 months 2 weeks	~ 1 year 2 months
	2	~ 4 days 12 h	~ 14 months	3	~ 11 days	~ 14 days
30 cores	114	~ 1 week	~ 1 months 2 weeks	114	~ 4 days 12 h	~ 4 weeks
	2	~ 4 h	~ 14 days	3	~ 9 h	~ 11 h
4032 cores	2	~ 1 min	~ 2 h 30 min	3	~ 4 min	~ 5 min

The duration times were effectively measured with a 4,032-cores-supercomputer and theoretically estimated for the others.

### Comparison between WRF and U-Net results

The F-Scores obtained between the segmentation obtained with U-Net and WRF methods were very similar for both staining and conditions as seen in Table 8 (1.2% differences in average for the optimized WRF). Therefore, the F-Score differences between U-Net and the optimized WRF with feature selection are comparable with the F-Score differences between optimized WRF without selection. The learning curves for U-Net method are presented in Figs. 7S and 8S for both staining. For anti-Phox2B, the F-Scores remained under 0.7. The learning time for each condition was around 45 min for NeuN_1 and 22 min for anti-Phox2B considering 1,000 epochs. The time differences were due to the difference in size of the two datasets: NeuN_1 datasets were twice the size of anti-Phox2B ones.

**Table 8:** Comparison between U-Net and optimized WRF F-Scores with and without feature selection for both staining and conditions.

Validation conditions	U-Net after 1000 epochs		Optimized WRF without feature selection		Optimized WRF after feature selection	
	NeuN_1	Anti-Phox2B	NeuN_1	Anti-Phox2B	NeuN_1	Anti-Phox2B
Direct	0.89	0.67	0.88	0.65	0.86	0.51
Cross	0.9	0.59	0.89	0.58	0.87	0.4

## Part III: Discussion

This study proposes a methodology to select a compact, robust and efficient features vector that can be used to perform image classification at large scale on massive histological images. An original two steps strategy was proposed: feature families’ selection, an original way of pooling features with similar sizes (step 1) and individual features selection, an aggregation strategy based on the most stable features detected through iterative process (step 2). A new quantitative criterion (fMPV) aiming to ensure robustness in the selection procedure was proposed to select features based on their presence in the best segmentation results derived from a set of combinations using an adapted brute-force strategy. For the two stainings selected to test different organs from four healthy macaques (3 brains, 1 brainstem) and neuron staining specificity, the proposed methodology achieved a consistent selection between direct and cross validations. Among the four NeuN datasets, the proposed two-steps selection process allowed a consistent selection for each step and each validation condition. The segmentation quality of NeuN between U-Net and optimized WRF without selection was similar, justifying our choice to use WRF in this work. Then the proposed methodology allowed a massive reduction of computation time and memory cost with a small loss in segmentation quality compared with U-Net segmentation quality (Table 8). Therefore, the selected vectors were suited to perform exhaustive quantification on whole organs even for group studies. However, for anti-Phox2B staining which is particularly difficult to segment (limited specificity of this staining as shown in Supplementary Fig. 8S) even for DL methods such as U-Net, the WRF segmentation quality did not reach satisfying F-Score neither with initial vector nor with selected one (F-scores ~ 0,6). The objective of this part of the work was to objectively evaluate the potential of segmentation methods in extreme cases. Despite low F-scores obtained, it could be envisioned to exploit these results by focusing on the detection of a certain type of neuron. In this context, the accuracy of the segmentation is less important compared to the assessment of a global mapping describing the spatial distribution of the cells of interest^32^.

For both staining, the optimization of WRF maximal depth led to a monotone and continuous curve convergence to a plateau as shown in Fig. 6 left. Also, the variation of WRF class of interest weight for anti-Phox2B led to a discrete function with an optimum (Fig. 6b right). Thus, the variation of these hyperparameters of WRF had similar behaviour through optimization for both staining even with default hyperparameters^55^. On the contrary, Deep Learning methods have numerous hyperparameters to tune (number of layers, size of different filters, feature roots, number of epochs, etc.) and their tuning can have unpredictable effects on segmentation quality^56,57^ as seen for anti-Phox2B in Fig. 8S. Such effects built the “black box” image that numerous people have on DL methods. Therefore, WRF seemed well suited as a segmentation algorithm for anti-Phox2B despite its low segmentation quality. Optimization of WRF hyperparameters was necessary especially to process anti-Phox2B, a non-specific staining. Without optimization, the F-Scores of DV and CV were below 0.5. The final F-Scores for anti-Phox2B were 0.65 in DV and 0.58 in CV, which were significantly lower compared to NeuN F-Scores even with U-Net. These low F-Scores can be explained by several factors. Stained neurons by anti-Phox2B were uncommon and had a little pixel-size-radius. F-Score was therefore a non optimal criterion for this staining but allowed comparison between segmentation quality for two stainings^49^. However, a 3D-surface rendering, delimiting the region where anti-Phox2B neurons distribution spread, has been manually pinpointed, as the first step towards the identification of respiratory centers in the brainstem^32^. The comparison between this ground-truth region (detection of individual neurons) and the one generated by the automatic segmentation using Hausdorff distance would be more relevant to assess neuron detection quality. Furthermore, the anti-Phox2B dataset could be pre- or post-processed. For example, the resulting segmentation would benefit from an enhancement of contrast, a color normalization as preprocessings^58^. In addition, the result of the segmentation could be regularized using morphological mathematics operations or median filtering. Further work will consider the possible effect of pre- and post-processing on the selection and the overall quality of segmentation. Another possible lead is to enhance the number of features in the initial dataset or use data augmentation methodologies^59,60^. The extension of the learning datasets in both size and features promise further improvement of segmentation quality.

Feature families were proposed to divide feature spaces into subparts presenting similar cardinality and to allow comparison of different types of features. Feature families’ selection showed that linear color spaces (RGB and XYZ) were the most descriptive color spaces for the NeuN dataset (Table 3) which is a staining highly specific (high contrast between stained and unstained tissue). L*a*b* was the least informative colorimetric color space. This result is consistent with instability of nonlinear color space at low saturation level^15^. On the contrary, nonspecific staining anti-Phox2B benefited from nonlinear color spaces as shown in Table 4. Linear color spaces seemed suited for specific staining and nonlinear color spaces for unspecific staining. However, this observation must be confirmed with supplementary tests on other staining. For anti-Phox2B, b* is one of the most informative features selected as shown in the second selection step Table 6. Both staining had an increase of F-Score with blue hues: B from RGB, b* from L*a*b* and Z from XYZ. Among primary colors, blue hue is the most discriminative for brown color^61^. Therefore, the blue hue was discriminative for NeuN and anti-Phox2B (two brown stainings), which was coherent with previous works^62^.

Real parts of Gabor families were ranked in high position in our selection feature scheme for both staining and each dataset (Tables 3 and 4). Combined with its low computation complexity, Gabor filters appeared to be adapted to perform large image classification. LBP and Variance images had similar advantages. For both staining, both features were particularly adapted for the set goal of feature vector size minimization, reduction of computation time and size wise. As shown in Tables 5 and 6, LBP and Variance images were indeed part of the stable feature subset for 4 out of 5 datasets. In order to process other staining, they are relevant candidate features, providing a good compromise between robustness, computational complexity and cardinality. Although Haralick texture feature space is not suitable for Big Data applications due to its high computational complexity and cardinality, it was tested in our study to confirm its ability to reach the highest F-Score possible for both staining and each datasets. Moreover, we noticed that the quality of the segmentation improved when decreasing radius. Haralick with a 2.5 µm radius for NeuN and anti-Phox2B datasets were the most compact of Haralick families in the best results, as shown in Supplementary Figs. 1, 2, 3, 4, 9 and 10. Texture close to the pixel scale (micro-texture) was more discriminative than texture at the neuron scale (macro-texture). Haralick features were originally used to discriminate between areas presenting uniform textures^35,36^. Here, two kinds of texture were available: intra- and extra-cellular (micro- and macro-textures). A process kernel with a larger radius than a third of the neuron size would not allow to characterize the intra-cellular texture properly.

The use of join vectors and meta-features^63,64^ would benefit feature exploration by reducing redundancy (for example between B, Z and b* as shown in the results) and pre-selecting relevant color or textural properties for subsequent segmentation. Consequently, the proposed methodology will be able to explore more possible feature vectors by reducing the combinatorial space. However, the use of join vectors or meta-features could lead to interpretability issues by concealing relevant features with “garbage” features^22^ limiting the efficiency and quality of subsequent classification. Further work will consist in finding a heuristic and a vector size supremum to allow a deeper exploration with an adapted Brute-force selection. For example, computing the mean F-Score for a sampled combinations space at each different vector size possible will allow us to estimate a minimum vector size. It will be determined to produce a F-Score value close to the initial feature vector segmentation quality including all the features. Another work will consist in using this estimation to define a heuristic to sample the entire combinatory space. If the minimum vector size requires computation of a large number of combinations, the definition of an adapted sampling strategy will be necessary. The validation of this approach will be based on the convergence of the results to a stable state coupled with an improvement of the F-Score criterion. This sampling strategy will be inspired by a multi-scale strategy proposed in the literature or genetic algorithms^29,46^. Finally, a feature family selection will be performed on various histology staining and animal species to make it possible to associate specific feature families to histological staining. The results will possibly provide guidelines to design pre-optimized initial feature vectors for various biological studies.

Using fMPV criterion resulted in similar feature sets selection for each staining in both DV and CV. Uniform pattern LBP and Variance images can be considered as an edge detector and Gabor filters real parts as a blob detector^65^. Both staining needed LBP or Variance image with a blob detector (real part of Gabor filters) to detect neurons. NeuN feature selection provided a feature vector with a high segmentation quality achieving a F-Score close to the initial feature vector including 114 features. Edge detectors were not selected for NeuN_3 alone. However, the selected vector of NeuN_3 reached a mean F-Score superior to 0.87 with only blob detectors which was the second highest F-Score through all selected vectors. For the other NeuN datasets, Mean image did not have a high fMPV and were discarded during the second selection step. As presented in Fig. 8, the selected vectors were located in the best combinations of NeuN_pool. Solely relevant features were selected in the first step of the methodology as presented in Fig. 7. Therefore, all combinations provided F-Scores superior to 0.8 which can explain the disparity in the final feature vectors through all NeuN datasets as multiple vectors fit the goal of the selection with similar performances. The anti-Phox2B selected feature vector was part of the top 14% of the combinations. However, it did not allow to reach such high F-Scores (in average, 0.45 against 0.61 for the initial vector, between DV and CV). The selection did not reach a F-Score similar to the F-Score with 114 features, meaning that the vector size limit was too low and would require more features. The extension of the number of feature spaces—such as wavelet transforms—can potentially improve the segmentation quality by taking into account multi- resolution. The ending condition proposed was adapted for specific staining but exhibits strong limitations on non-specific staining. Further work will aim to define new ending conditions depending on the F-Scores and their distribution. This study demonstrated the relevance of the presented methodology for mono- (anti-Phox2B) and multi-subjects (NeuN) studies. In each case, similar vectors were selected for a specific subject and, in the case of NeuN datasets, all selected vectors derived from similar features and were highly ranked with fMPV. Further studies will be conducted to extract relevant feature vectors on publicly available H&E databases such as TCGA^66^.

On a more general note, the proposed methodology is part of the Wrapper methods—selection process including learning and validation steps^67,68^—and Brute-force searching is the weakest of all meta-heuristic. Consequently, the other meta-heuristic results can be compared to Brute-Force searching ones as reference to other FSA. Further research should lead us to benchmark our methodology with other feature selection methods such as correlation filters, join vectors, genetic algorithms, minimum-redundancy-maximum-relevance feature selection or PCA. Such study is difficult to achieve due to the large number of FSA and ML algorithms to explore, control and observe^23^. However, with the use of Brute-force searching, the feature selection algorithm will be compared through stability of selection in addition with the traditional parameters such as segmentation quality and number of features selected. These methods will be compared through: (1) stability, (2) F-Score computed with the selected subset, (3) computational complexity and (4) memory requirements.

Our adapted Brute-force searching computation approach was costly in terms of computation resources. However, computational time and memory needed were drastically decreased due to the strong scalability of our method. Indeed, feature extraction, classification and Brute-Force selection were highly parallelizable. Moreover, the selection process to reduce features to be considered for segmentation was worth in terms of consumption time even in only one entire digitized section processed. In a more general perspective, reduction of computational complexities or reduction of high-extraction-time features should be one of the primary focuses to design a machine learning workflow. In fact, the energetic consumption of HPC infrastructures coupled with physical limitations of semiconductors will strongly constrain HPC hardware progress^69,70^. Optimization and rationalization of computing resources and methodologies will become mandatory to pursue dealing with Big Data.

In the biomedical image analysis field, the significant reduction in computation time and memory allow exhaustive quantification and analysis of any massive images where classical analyses are limited to a small number of histological sections and in a restricted set of small regions of interest. The proposed method extends the amount of images to be processed in a short period of time and therefore increases our ability to carry out ambitious group studies^5^.

## Conclusion

The proposed methodology allowed us to select small and stable feature vectors for histological section segmentation. The selected feature vectors made possible the processing of high-resolution images (2 whole stained organs) by reducing time and memory consumption. A combination between edge and blob detectors was relevant for biological object segmentation, linear color spaces for specific staining (NeuN) and non-linear color spaces for non-specific staining (anti-Phox2B). Textural information was mandatory and particularly relevant to reach high F-Score for pixel scale segmentation in histological images. Ongoing work is focusing on the improvement of the proposed methodology to allow deeper exploration and selection, by extending the feature vector size limit through sampling of combination space. Another work will consist in comparing Brute-Force selection with other FSA. The main goal is to propose generic guidelines for FSA highlighting their assets and drawbacks. Moreover, a fully industrialized platform based on HPC cloud computing will be implemented, which will decrease the time necessary for preclinical and clinical group studies.

## Supplementary Information

Supplementary Information.
