# Supplementary material for: Reduced and stable feature sets selection with random forest for neurons segmentation in histological images of macaque brain
Source: Sci Rep. 2021 Nov 26;11:22973. doi: 10.1038/s41598-021-02344-6 (PMC8626511; doi:10.1038/s41598-021-02344-6)
Supplement: Supplementary file 1 — Supplementary Information. [file 41598_2021_2344_MOESM1_ESM.docx]

**Reduced and stable feature sets selection with Random Forest for neurons segmentation in histological images of macaque brain**

C. Bouvier^1,2^, N. Souedet^1^, J. Levy^3,4^, C. Jan^1^, Z. You^1,5^, A-S Herard^1^, G. Mergoil^2^, B.H. Rodriguez^2^, C. Clouchoux^1,2^, T. Delzescaux^1,*^

1 Université Paris-Saclay, CEA, CNRS, MIRCen, Laboratoire des Maladies Neurodégénératives, Fontenay-aux-Roses, France

2 Witsee, Paris, France

3 Service de Médecine Physique et de Réadaptation – APHP Hôpital Raymond Poincaré, Garches

4 UMR 1179, Handicap Neuromusculaire – INSERM-UVSQ, Montigny le Bretonneux

5 School of Computer Science and Engineering, Xi’an University of Technology, Xi’an, China

*[thierry.delzescaux@cea.fr](mailto:thierry.delzescaux@cea.fr)

**Supplementary data:**


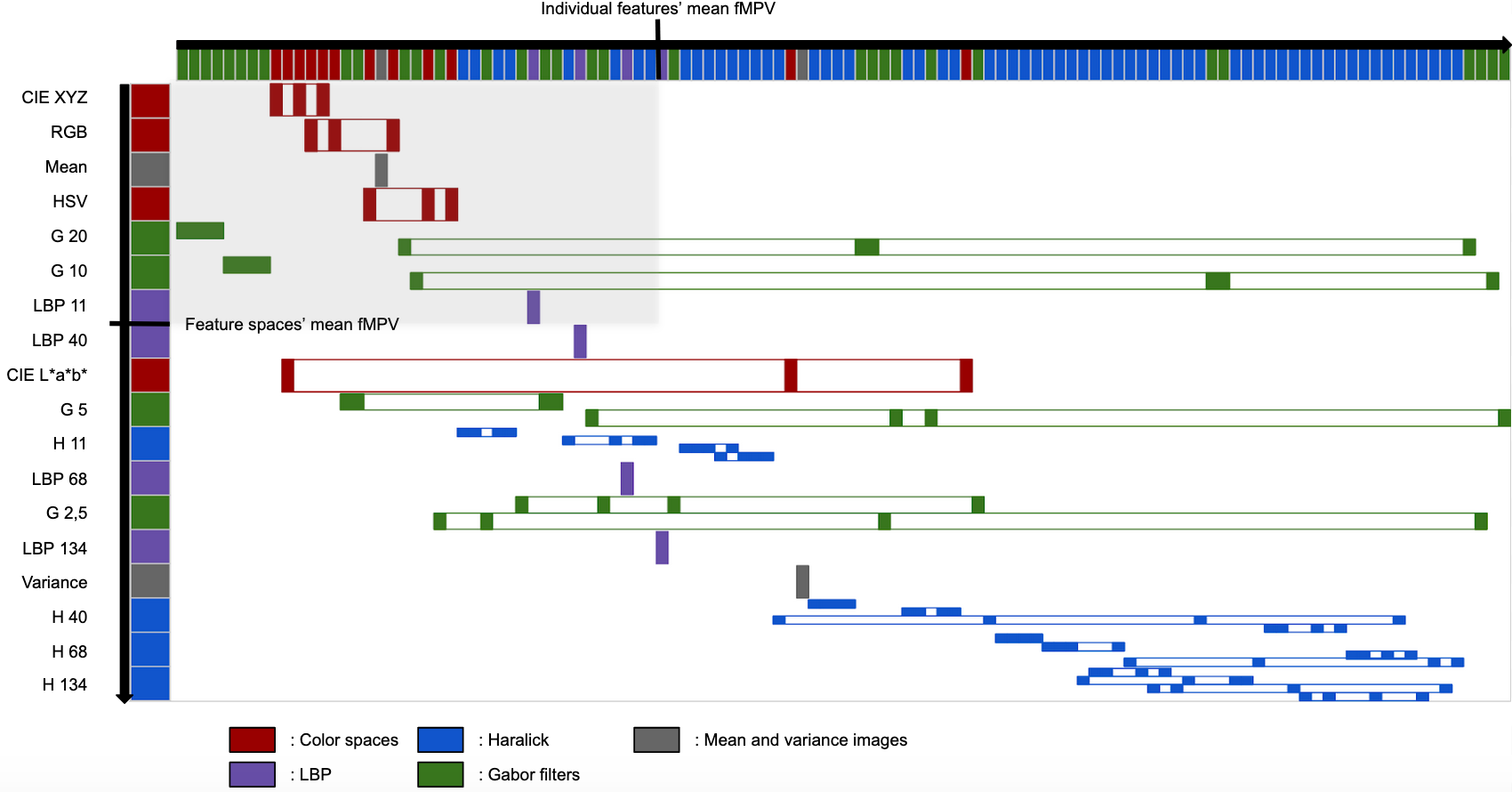


Figure 1S: First step for NeuN_1 in DV. The horizontal axis represents the ranking through fMPV of the 114 individual features (the best features on the left and the worst on the right). The vertical axis represents the ranking through fMPV of the feature spaces (the best spaces on the top and the worst on the bottom). Feature families are represented through these axes. The selected feature families for NeuN_1 in Direct Validation are highlighted in a grey frame.


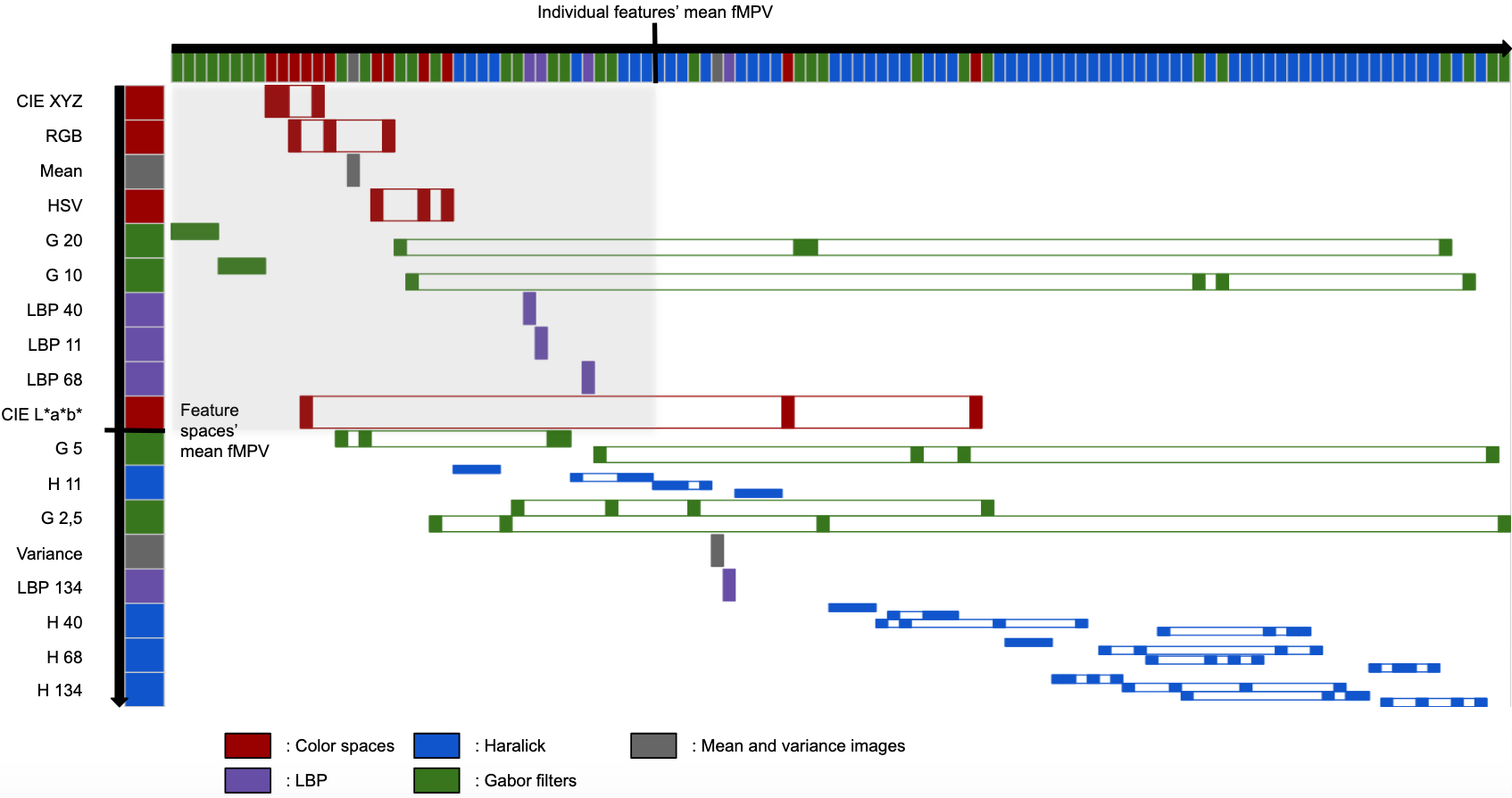


Figure 2S: First step for NeuN_1 in CV. The horizontal axis represents the ranking through fMPV of the 114 individual features (the best features on the left and the worst on the right). The vertical axis represents the ranking through fMPV of the feature spaces (the best spaces on the top and the worst on the bottom). Feature families are represented through these axes. In the grey square are highlighted the selected feature families for NeuN_1 in Cross Validation.


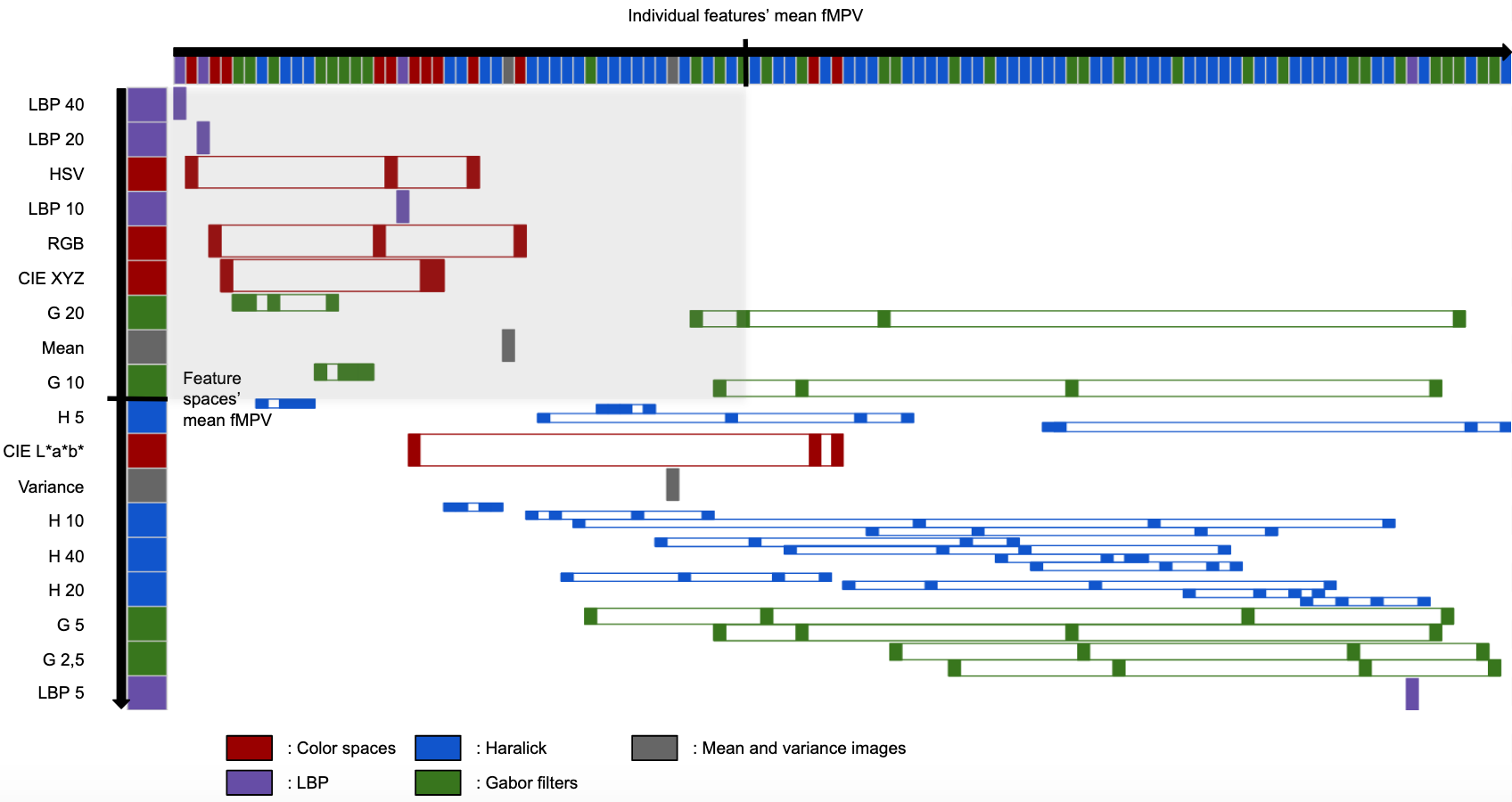


Figure 3S: First step for anti-Phox2B in DV. The horizontal axis represents the ranking through fMPV of the 114 individual features (on the left were the best features and on the right the worst). The vertical axis represents the ranking through fMPV of the feature spaces (the best spaces on the top and the worst on the bottom). Feature families are represented through these axes. In the grey frame are the selected feature families for DAB anti-Phox2B in Direct Validation.


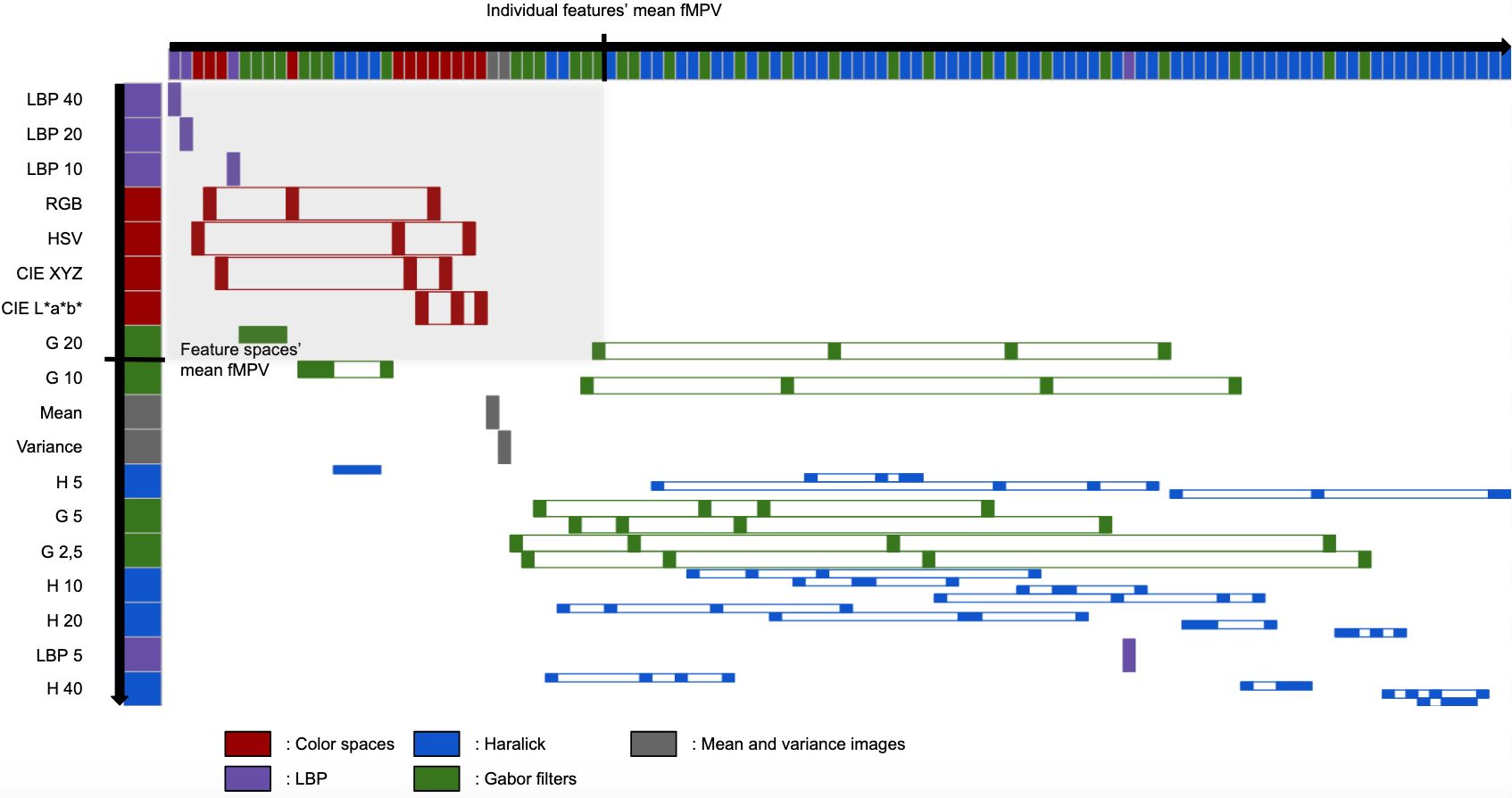


Figure 4S: First step for anti-Phox2B in CV. The horizontal axis represents the ranking through fMPV of the 114 individual features (on the left were the best features and on the right the worst). The vertical axis represents the ranking through fMPV of the feature spaces (the best spaces on the top and the worst on the bottom). Feature families are represented through these axes. In the grey frame are the selected feature families for DAB anti-Phox2B in Cross Validation.


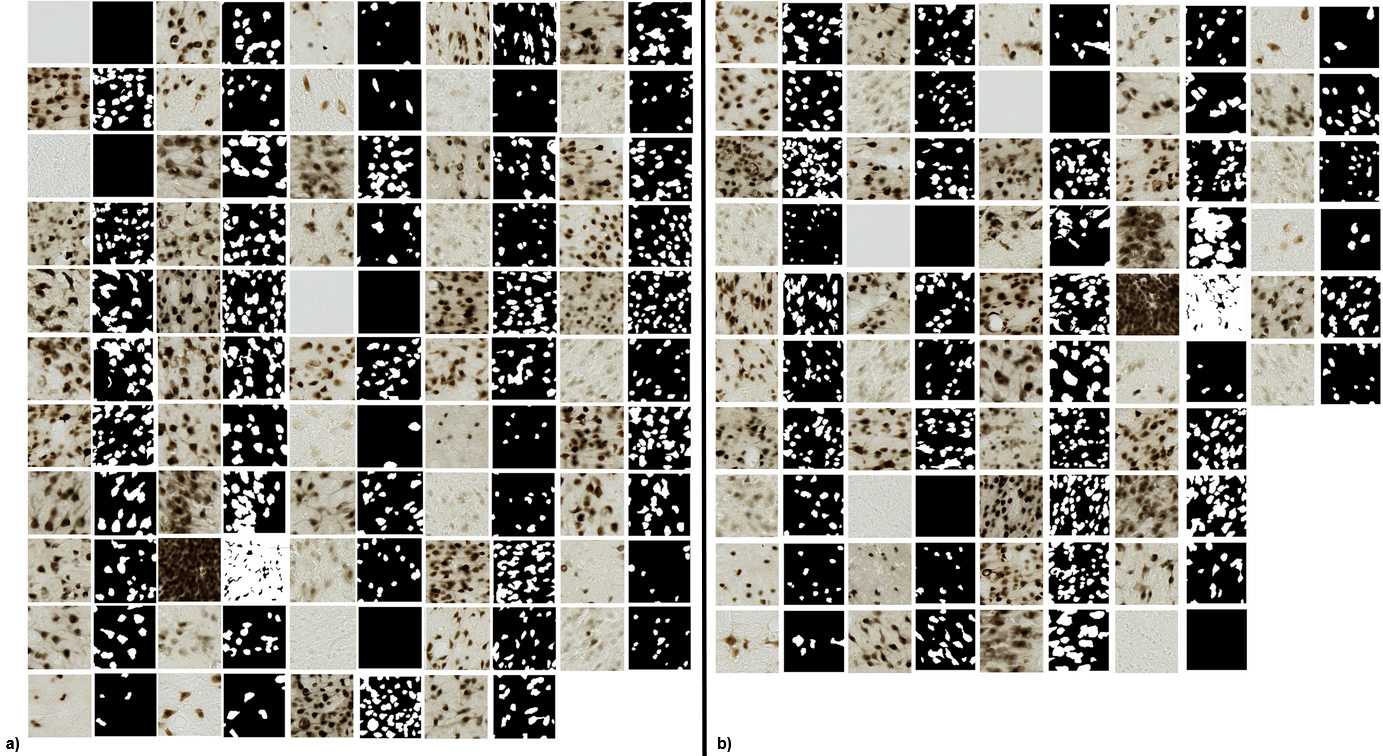


Figure 5S: Example of dataset used for DAB-Ni NeuN study (NeuN_1). DV condition: **a)** learning dataset and **b)** test dataset.


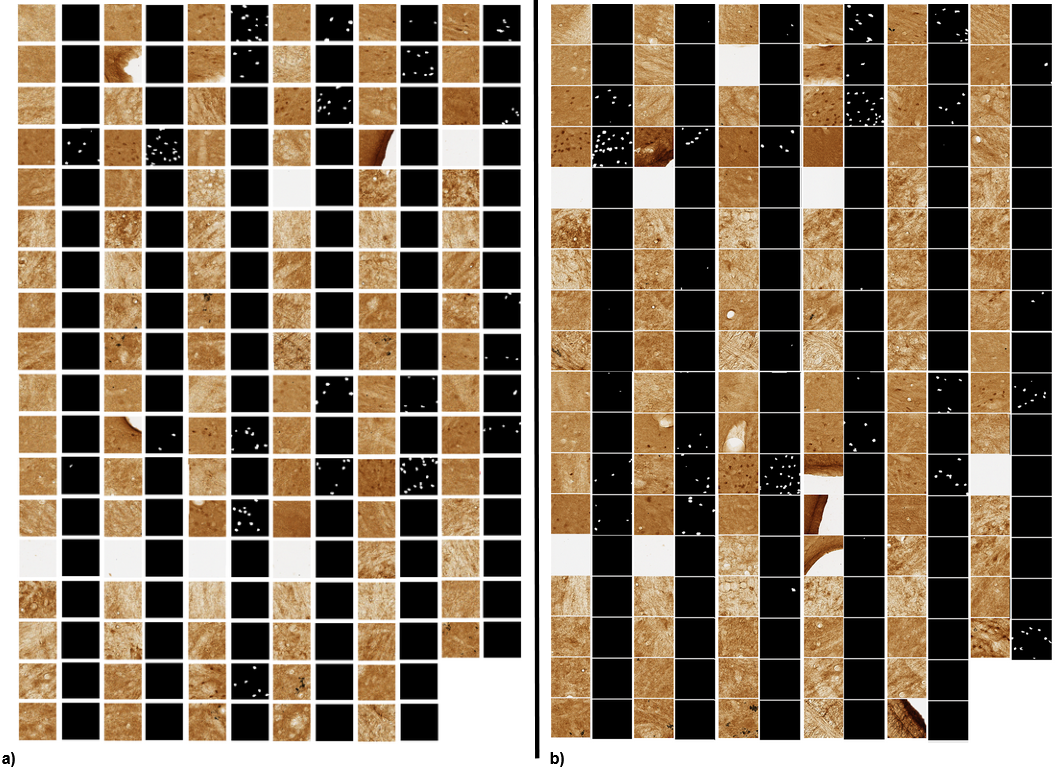


Figure 6S: Dataset used for DAB anti-Phox2B study. DV condition: **a)** learning dataset and **b)** test dataset.


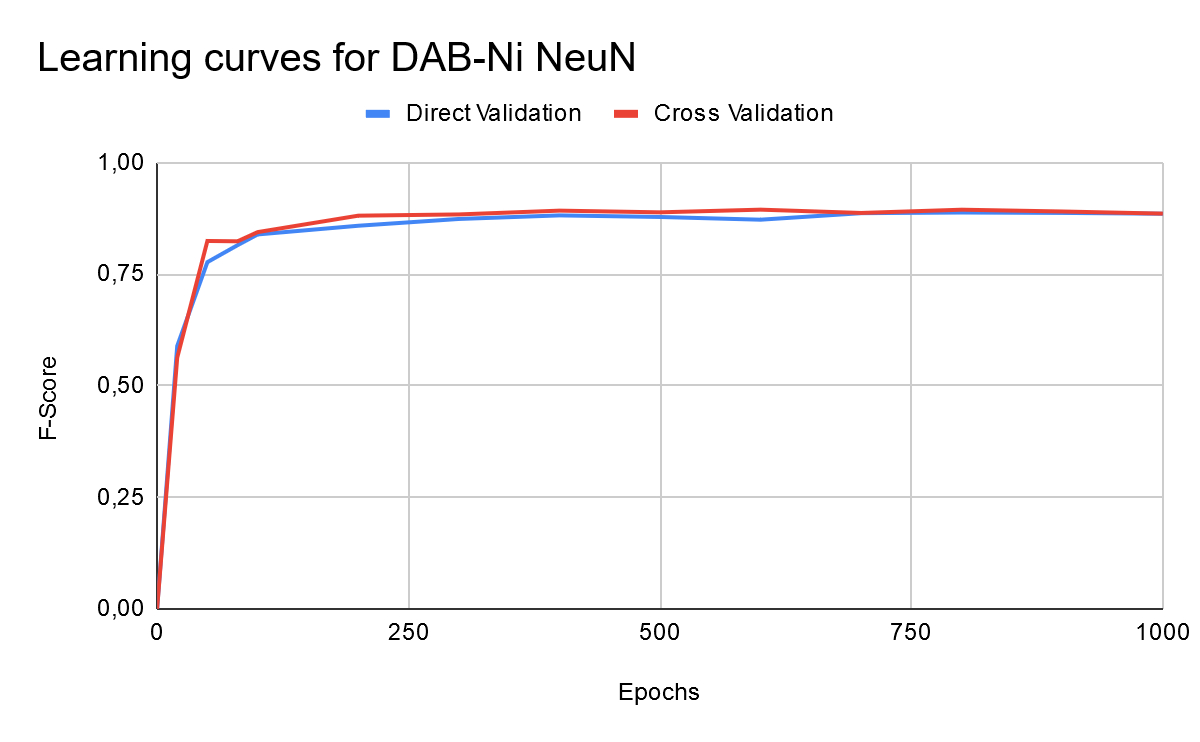


Figure 7S: F-Scores computed according to the number of epochs for DAB-Ni NeuN.


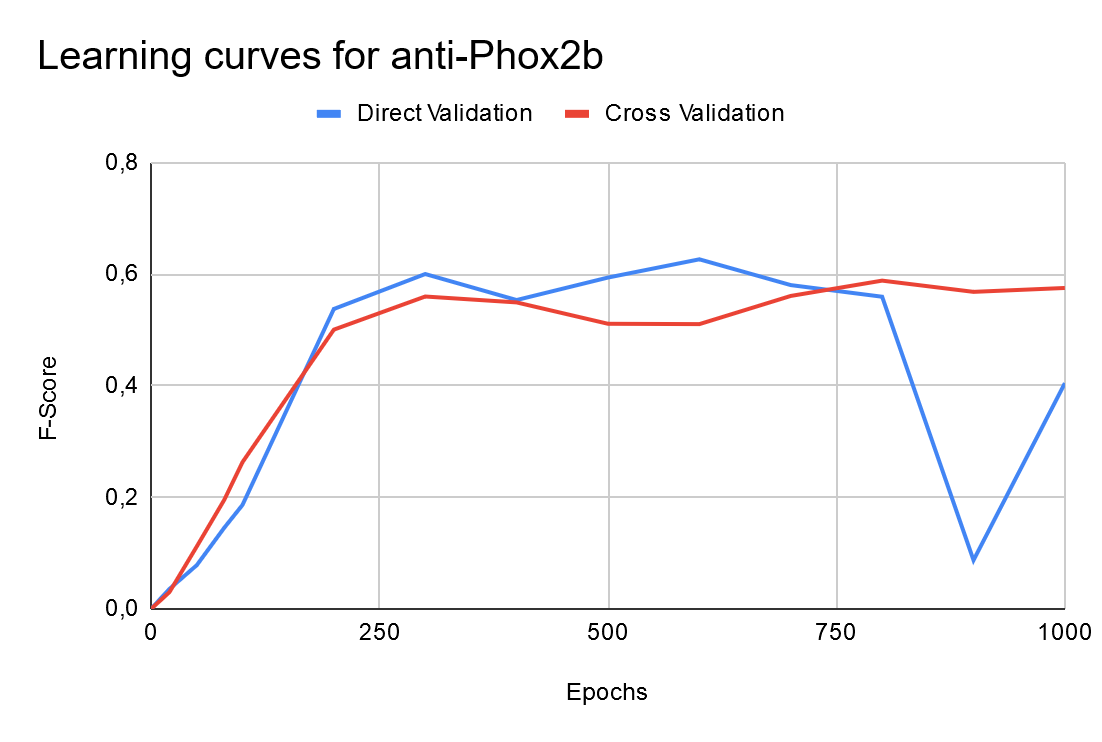


Figure 8S: F-Scores computed according to the number of epochs for DAB anti-Phox2B.


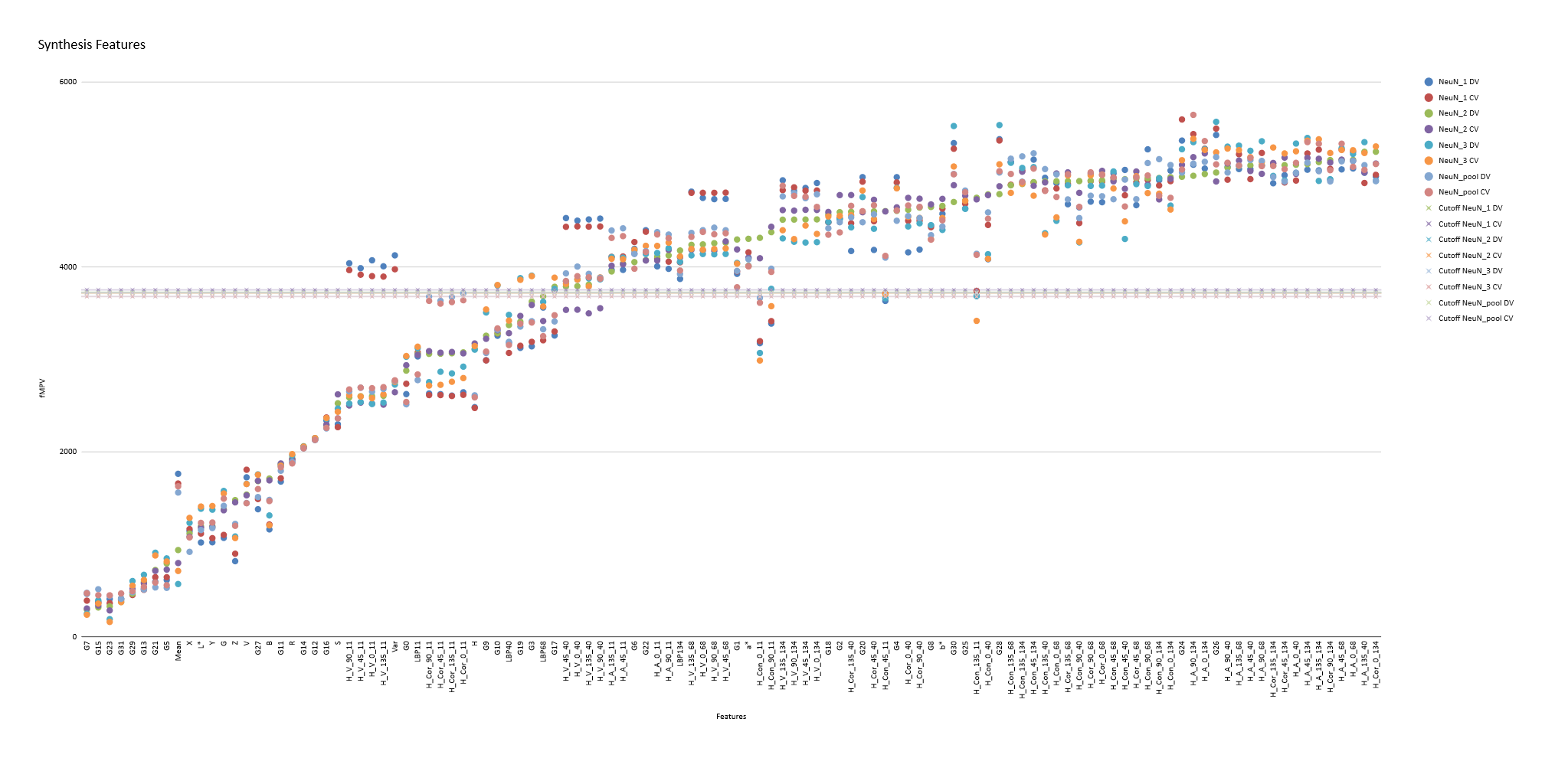


Figure 9S: Distribution of the 114 initial features’ fMPV for the four NeuN datasets. The different cutoffs are represented with cross points.


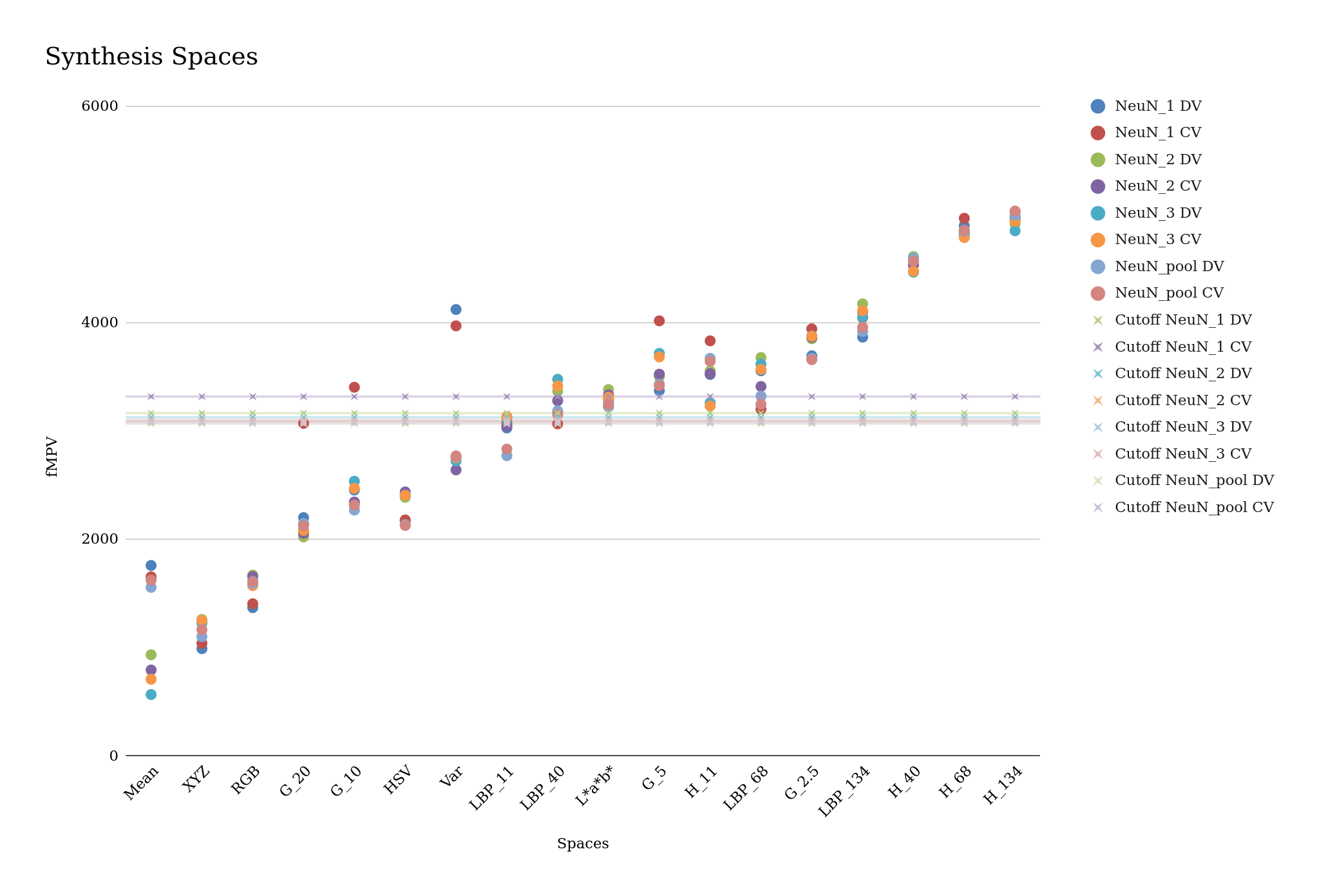


Figure 10S: Distribution of 18 initial spaces’ fMPV for the four NeuN datasets. The different cutoffs are represented with cross points.


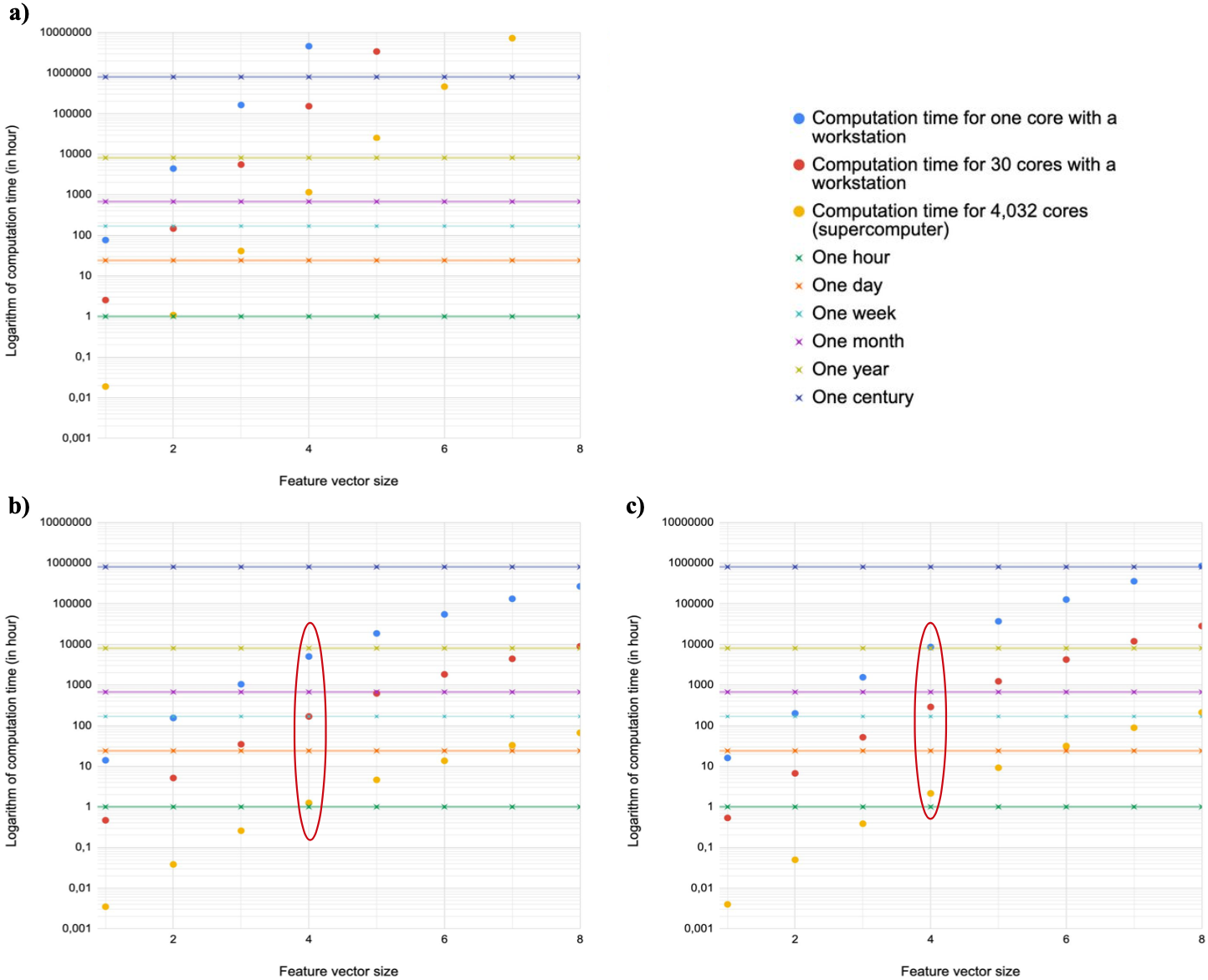


Figure 11S: Computation time (logarithmic scale) for different number of features and different computation settings. The horizontal lines represent different time scales (from an hour to a century) and the computation time of a combination was considered to be equal to 40 minutes. **a)** Computation time for 114 features, **b)** computation time for 21 features ( NeuN_1) and **c)** computation time for 24 features (DAB anti-Phox2B). The circled points represented reasonable processing times for the three different computation configurations with a maximal vector size of 4 in b) and c).
